# Regular exercise ameliorates high-fat diet-induced depressive-like behaviors by activating hippocampal neuronal autophagy and enhancing synaptic plasticity

**DOI:** 10.1038/s41419-024-07132-4

**Published:** 2024-10-10

**Authors:** Jialin Wu, Huachong Xu, Shiqi Wang, Huandi Weng, Zhihua Luo, Guosen Ou, Yaokang Chen, Lu Xu, Kwok-Fai So, Li Deng, Li Zhang, Xiaoyin Chen

**Affiliations:** 1 School of Traditional Chinese Medicine, Jinan University, Guangzhou, China; 2 Key Laboratory of Central CNS Regeneration (Ministry of Education), Guangdong-Hong Kong-Macau Institute of CNS Regeneration, Jinan University, Guangzhou, China

**Keywords:** Depression, Depression

## Abstract

Exercise enhances synaptic plasticity and alleviates depression symptoms, but the mechanism through which exercise improves high-fat diet-induced depression remains unclear. In this study, 6-week-old male C57BL/6J mice were administered a high-fat diet (HFD, 60% kcal from fat) to a HFD model for 8 weeks. The RUN group also received 1 h of daily treadmill exercise in combination with the HFD. Depressive-like behaviors were evaluated by behavioral assessments for all groups. The key mediator of the effect of exercise on high-fat diet-induced depressive-like behaviors was detected by RNA-seq. The morphology and function of the neurons were evaluated via Nissl staining, Golgi staining, electron microscopy and electrophysiological experiments. The results showed that exercise attenuated high-fat diet-induced depressive-like behavior and reversed hippocampal gene expression changes. RNA-seq revealed Wnt5a, which was a key mediator of the effect of exercise on high-fat diet-induced depressive-like behaviors. Further work revealed that exercise significantly activated neuronal autophagy in the hippocampal CA1 region via the Wnt5a/CamkII signaling pathway, which enhanced synaptic plasticity to alleviate HFD-induced depressive-like behavior. However, the Wnt5a inhibitor Box5 suppressed the ameliorative effects of exercise. Therefore, this work highlights the critical role of Wnt5a, which is necessary for exercise to improve high-fat diet-induced depression.

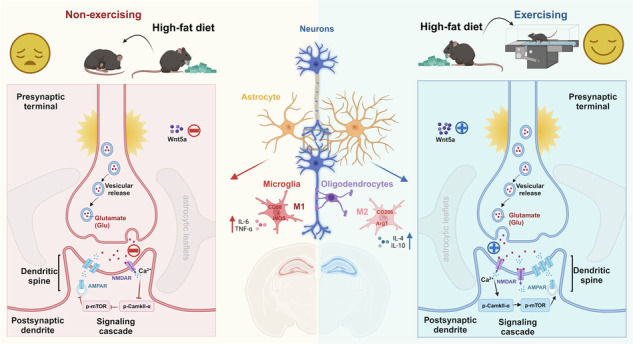

## Introduction

High-fat diet (HFD) consumption, a highly prevalent lifestyle behavior, is acknowledged as a substantial risk factor for noncommunicable diseases [1] and has insidious, cumulative, and progressive adverse effects [2]. Whether HFD causes psychiatric disorders is related to duration of dietary exposure, age, species, and genetic background [3]. It has been shown that HFD consumption in young animals for a short period of time can provide saturated long-chain fatty acids necessary for myelin generation, and enhance neuroplasticity, which is beneficial for improving learning and memory ability [4, 5]. However, converging evidence from clinical investigations, epidemiological studies, and animal experiments consistently reveals a compelling association between long-term HFD consumption and increased susceptibility to cognitive and emotional impairments [6–10]. Although the brain requires fatty acids to maintain normal function, long-term consumption of HFD affects metabolism and immunity [11, 12] which can reduce hippocampal volume, and it also has the potential to impair the function of neurons and glial cells [13, 14], impairing cognitive function and increasing vulnerability to depression [8, 15]. Nonetheless, the biological mechanisms responsible for the neurobehavioral alterations triggered by HFD consumption have not been fully elucidated.

Notably, increasing evidence suggests that HFD consumption may impair autophagy in diverse peripheral tissues, including the liver, cardiac muscle, skeletal muscle, and adipose tissue, thereby influencing the trajectory of associated pathologies [16–19]. Similarly, HFD consumption can suppress neuronal autophagy within the cerebral milieu, resulting in the inhibition of autophagic processes in hypothalamic, hippocampal, and prefrontal cortical neurons and culminating in disturbances in appetite, cognition, and emotions [20–23]. Defects in autophagy lead to the accumulation of ubiquitin-positive protein aggregates, axon swelling, and neuronal degeneration [24], whereas autophagy-inducing drugs have antidepressant-like properties in mice [25], and many clinically prescribed antidepressants with different pharmacological activities enhance autophagy [26]. Additionally, multiple studies have shown that autophagy plays a role in the structural recombination of neuronal circuits through axon growth, dendritic spine formation and pruning, synaptic assembly, and vesicular turnover [27, 28].

In concordance with conventional wisdom, several studies have substantiated the beneficial effects of regular physical exercise in preventing or ameliorating a spectrum of systemic conditions induced by HFD consumption, especially metabolic and neurological diseases [29–31]. Physical exercise, an established strategy for preventing depression, has demonstrated a substantial capacity to avert or mitigate depressive symptoms and cognitive impairments [32–35]. Exercise, a nondrug physiological therapy, may effectively activate autophagy in a short period [36], which is conducive to maintaining tissue integrity, inhibiting the inflammatory response, controlling tissue damage or activating direct signaling pathways to adapt to physiological changes in the body [37]. Numerous studies have shown that exercise-induced changes in autophagy capacity, including increased autophagy flux and activation of key autophagy gene transcription, may lead to enhanced autophagy activity [38, 39]. Generally, autophagy is mediated by many different signaling pathways, mainly including mTOR kinase inhibition [27, 38]. Interestingly, evidence has demonstrated that exercise inhibits mTOR signaling, further contributing to endurance exercise-induced autophagy [37, 38]. Similarly, some previous studies by our group showed that treadmill training could regulate the mTOR pathway to enhance synaptic plasticity in cortical neurons, thereby improving learning and memory ability and mental stress resistance [40–42]. However, the complete cellular signaling mechanism by which exercise alleviates HFD-induced depression-like behavior has not been elucidated, especially the molecular mechanism underlying the upstream mechanism of motor regulation of mTOR.

In summary, we hypothesize that exercise training may mediate the mTOR pathway and thus affect hippocampal synaptic plasticity, thereby alleviating HFD-induced depression-like behavior. To test this hypothesis, we successfully established a high-fat diet-induced depression-like behavior model by feeding adult mice a high-fat diet for 8 weeks. In contrast, treadmill exercise combined with a high-fat diet effectively ameliorates depression-like behavioral phenotypes by improving hippocampal synaptic plasticity, which depends on Wnt5a/CamkII/mTOR signaling pathway-mediated autophagy activation. Further molecular dissection revealed the role of Wnt5a, which positively affects neuronal autophagy mediated by the CamkII/mTOR pathway under HFD conditions. In summary, the Wnt5a/CamkII/mTOR signaling pathway identified in this study provides new insights into how exercise alleviates HFD-induced depression-like behaviors.

## Results

### Physical exercise alleviates high-fat diet-induced depressive-like behaviors and damage to hippocampal neurons

**Fig. 1:**
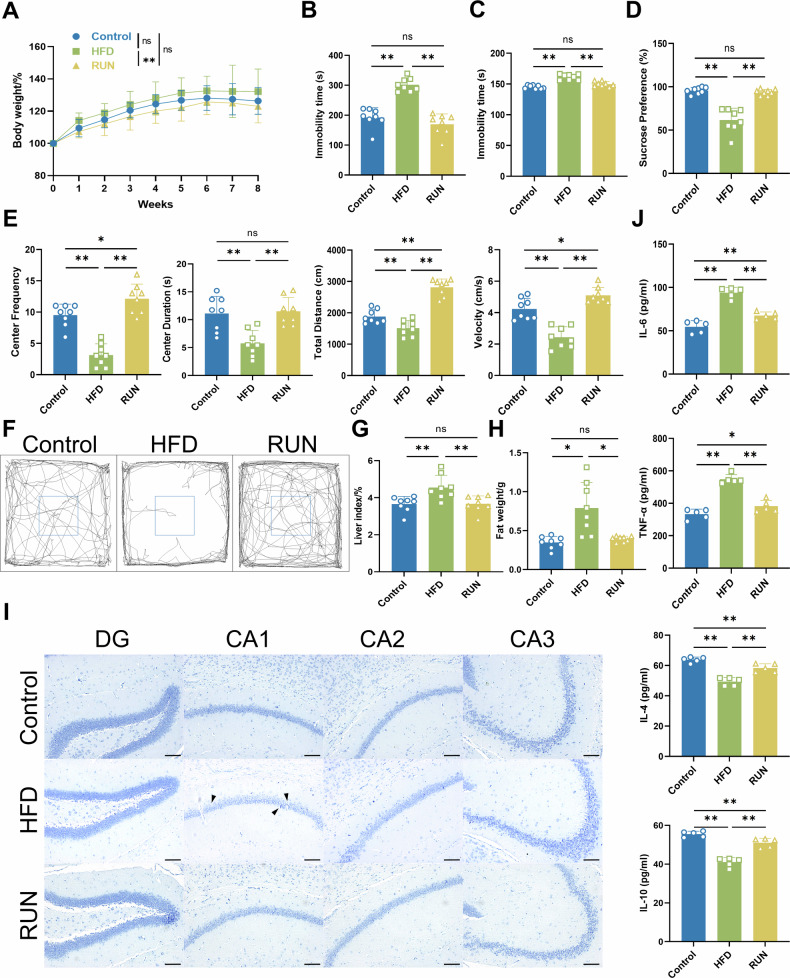
Ameliorative effects of exercise on depressive-like behavior and hippocampal neuronal damage induced by a high-fat diet. **A** Changes in the body weights of the mice (*n* = 12). **B** Immobility time in the tail suspension test (TST) (*n* = 8). **C** Immobility time in the forced swimming test (FST) (*n* = 8). **D** Sucrose preference test (*n* = 8). **E** Comprehensive behaviors of the mice in the open field test (OFT) (*n* = 8). **F** Track visualization image of the OFT. **G** Effect of exercise on the liver indices (*n* = 8). **H** Effect of exercise on epididymal fat weight (*n* = 8). **I** Influence of exercise on the arrangement and number of hippocampal neurons in mice fed a high-fat diet (bar = 100 μm). The black arrows indicate disorganized neurons. **J** Exercise effects on high-fat diet-induced neuroinflammation (*n* = 5). All the results are presented as the means ± standard deviations (SDs) with statistical significance (*P < 0.05, **P < 0.01, ns: *P* > 0.05).

To investigate whether concurrent exercise in combination with a high-fat diet has a beneficial effect on preventing depressive-like behaviors, we first established a mouse model of depression; mice in the HFD group and the RUN group were both fed a HFD, whereas those in the Control group were not. The detailed experimental timeline is presented in Fig. S1. After 8 weeks, the average body weight of the mice in the HFD group was heavier than that of the mice in the Control group, and exercise significantly reduced the body weight of the mice (Fig. 1A). In the tail suspension test and the forced swimming test, mice in the HFD group exhibited a significant increase in immobility time, whereas the immobility time was significantly reduced in the RUN group (Fig. 1B, C). In the sucrose preference test, exercise restored the preference of mice fed a high-fat diet for sucrose water (Fig. 1D). The trajectories of the mice in the open field test were analyzed, and we observed that the number of entries into the center zone, the time spent in the center zone, the total distance traveled and the velocity were significantly lower in the HFD group than in the Control group, while exercise obviously reversed the effects of high-fat diet feeding (Fig. 1E, F). Exercise restored locomotor activity and exploratory behavior in the mice. Behavioral analysis has shown that chronic consumption of a high-fat diet leads to depressive-like behaviors in mice and that exercise is effective at preventing depressive mood induced by a high-fat diet [43–45]. After behavioral assessment, it was found that although high-fat diet did not have a significant effect on body weight, it dramatically increased the body size, liver indices, epididymal fat weights and serum lipids; these effects were markedly inhibited by exercise (Figs. 1G, H and S2A, B). Along with preventing depressive-like behaviors, exercise also ameliorated the high-fat diet-induced disorganization of hippocampal neurons and the reduction in the number of neurons in the hippocampus, which was particularly pronounced in the hippocampal CA1 region (Fig. 1I). In addition, exercise significantly reduced neuroinflammation in the mouse hippocampus (Fig. 1J).

### Exercise regulates hippocampal gene expression in HFD-fed mice

**Fig. 2:**
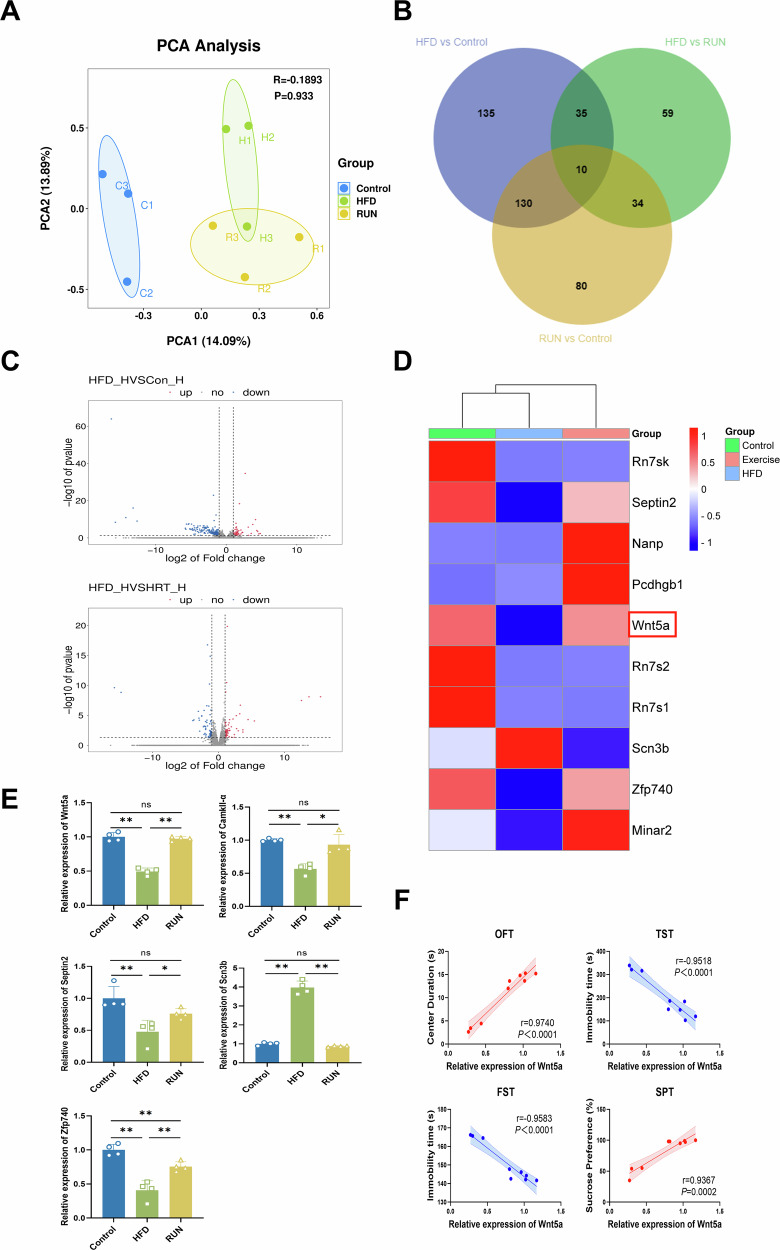
Exercise regulates hippocampal gene expression in HFD-fed mice. **A** PCA plot (*n* = 3). **B** Venn diagram. **C** Volcano plots of differential gene expression levels between groups (Con_H: the Control group; HFD_H: the HFD group; HRT_H: the RUN group). Significantly differentially expressed genes are shown in different colors; the blue dots indicate downregulated genes, and the red dots indicate upregulated genes. **D** Heatmap of the top 10 DEGs (Control: the Control group; HFD: the HFD group; Exercise: the RUN group). **E** Results of PCR validation (*n* = 4). **F** Correlation analysis of Wnt5a expression with the results of behavioral tests (*n* = 3). All the results are presented as the means ± standard deviations (SDs) with statistical significance (*P < 0.05, **P < 0.01, ns: *P* > 0.05).

After observing the ameliorative effect of exercise on depressive-like behavior in mice, we obtained hippocampal tissues for RNA-seq. The results showed that exercise altered the expression of hippocampal genes in mice. According to the PCA plot, samples from the same group were clustered, whereas samples from the different groups were separated, revealing a significant difference in gene expression (Fig. 2A). Venn diagrams revealed a total of 310 genes that were differentially expressed between the Control and HFD groups and 138 genes that were differentially expressed between the HFD and RUN groups (Fig. 2B). A volcano plot revealed that, compared with those in the Control group, a total of 92 genes were upregulated and 218 genes were downregulated in the HFD group. Compared with those in the RUN group, 69 genes in the HFD group were upregulated, and 69 genes were downregulated (Fig. 2C). Among the DEGs in the three groups, the top 10 genes were Rn7sk, Septin2, Nanp, Pcdhgb1, Wnt5a, Rn7s2, Rn7s1, Scn3b, Zfp740, and Minar2 (Fig. 2D). In particular, exercise modulated the expression of 4 genes, namely, Septin2, Wnt5a, Scn3b and Zfp740. The expression of these genes in the hippocampus was changed in HFD-fed mice; however, they showed similar trends in the RUN group as in the Control group. The results were verified by PCR and were in accordance with the RNA-seq results (Fig. 2E).

To identify the gene that is primarily regulated by exercise, we correlated the expression data for these 4 genes with behavioral data, including OFT, TST, FST and SPT data. We found that the expression of Wnt5a had the most obvious linear correlation with performance among these behavioral tests (Figs. 2F and S3A). GO enrichment analysis of the identified genes is shown in Fig. S3B. Wnt5a regulates axonal and dendritic growth as well as synapse generation in hippocampal neurons and is important for the long-term stability of hippocampal dendritic structures [46–49]. We explored how exercise exerts an antidepressant effect through Wnt5a in subsequent experiments.

### Exercise enhances synaptic transmission by activating the Wnt5a/CamkII signaling pathway

**Fig. 3:**
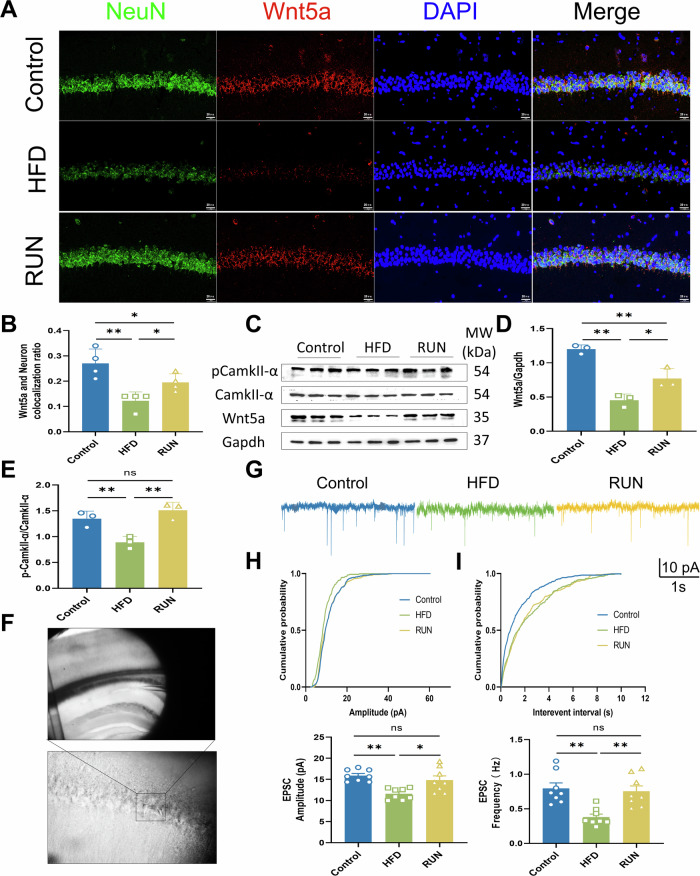
Effect of exercise on the Wnt/Ca^2+^ signaling pathway and synaptic transmission in HFD-fed mice. **A** Representative immunofluorescence image of neurons in the hippocampal CA1 region labeled with Wnt5a and NeuN (bar = 20 μm). **B** Statistical analysis of the ratio of Wnt5a to NeuN colocalization (*n* = 4). **C** Representative images of the western blot and **D** relative Wnt5a protein expression levels and **E** protein expression levels of p-CamkII-α/CamkII-α in the different groups (*n* = 3). Gapdh served as control. Full and uncropped western blots are detailed in the supplementary material. **F** Electrophysiological schematic. **G** Sample traces of excitatory postsynaptic current (EPSC). **H** Cumulative probability and comparison of the means of the EPSC amplitude (*n* = 8 from 4 mice in each group). **I** Cumulative probability and mean EPSC frequency (*n* = 8 from 4 mice in each group). All the results are presented as the means ± standard deviations (SDs) with statistical significance (*P < 0.05, **P < 0.01, ns: *P* > 0.05).

The RNA-seq results showed that the expression level of Wnt5a was reduced in the HFD group, whereas exercise modulated the expression of Wnt5a. The immunofluorescence results suggested that neurons with the highest percentage of colocalization with Wnt5a presented the greatest changes under the influence of high-fat diet (Figs. 3A, B and S4). Thus, exercise may exert an antidepressant effect by activating Wnt5a. Wnt5a supports the dendritic spines of pyramidal neurons in the CA1 region of the hippocampus through CamkII-mediated signaling, which mediates excitatory synaptic transmission [50]. Short-term sustained activation of CamkII-α is achieved through autophosphorylation of threonine 286 (Thr286), which mediates subsequent signaling cascades. As verified by Western blot analysis of hippocampal tissues, Wnt5a and p-CamkII-α levels were reduced in the HFD group, and these changes were reversed by exercise (Fig. 3C–E). According to sample electrophysiological recordings (Fig. 3G) of acute brain slices from the CA1 region of the hippocampus (Fig. 3F), the amplitude (Fig. 3H) and frequency (Fig. 3I) of excitatory postsynaptic currents were significantly altered in the exercise group compared with the model group, suggesting that synaptic transmission was enhanced.

### Exercise-induced activation of the Wnt signaling pathway promotes hippocampal neuronal autophagy and remodels synaptic structure

**Fig. 4:**
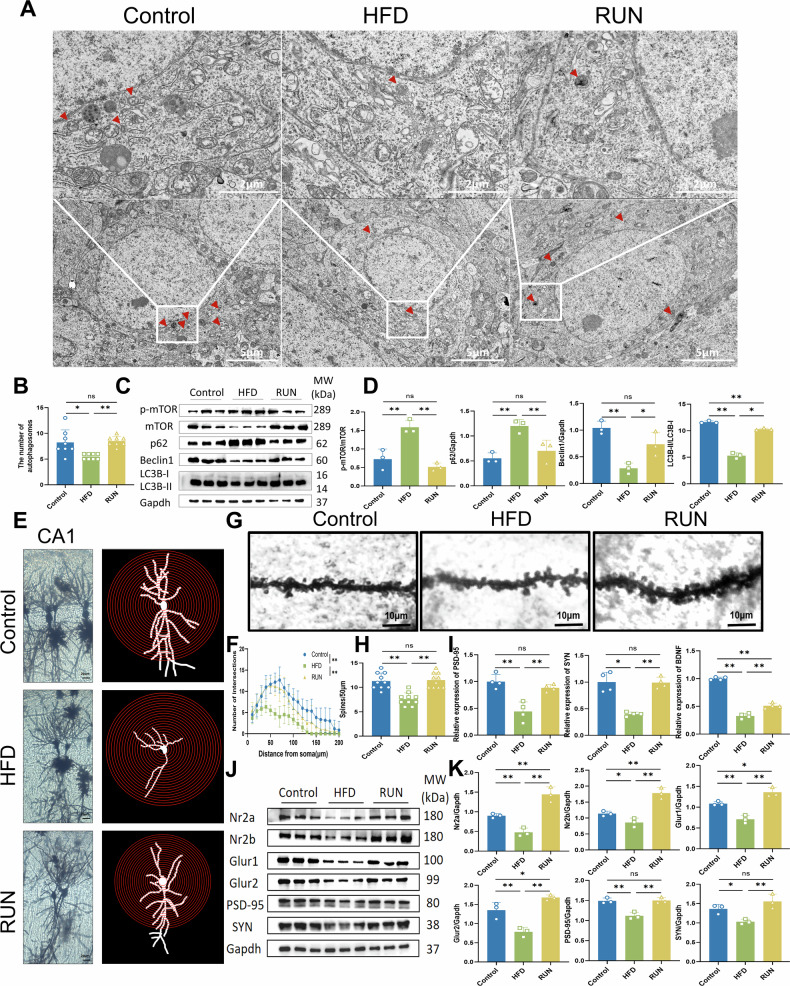
Exercise-induced activation of the Wnt signaling pathway promotes hippocampal neuronal autophagy and remodels synaptic structure. **A** Ultrastructure of hippocampal neurons under a transmission electron microscope (bar = 5 μm/2 μm). Red arrows indicate autophagosomes. **B** Statistical analysis of the number of autophagosomes (*n* = 8). **C** Representative images of the western blot and **D** relative expression of p-mTOR/mTOR, p62, Beclin 1 and LC3B (*n* = 3). Gapdh served as control. Full and uncropped western blots are detailed in the supplementary material. **E** Representative images of Golgi staining of neurons in the CA1 region of the hippocampus (bar = 20 μm). **F** Fuji Sholl analysis of the dendritic complexity of neurons in the CA1 region. Statistical analysis: two-way ANOVA followed by the Tukey post hoc test (*P < 0.05, **P < 0.01, ns: *P* > 0.05; *n* = 3 mice, 2 neurons from the CA1 region per mouse). **G** Representative images of Golgi-stained dendritic spines (bar = 10 μm). **H** Exercise increases dendritic spine density. Statistical analysis: two-way ANOVA followed by Tukey’s post hoc test (*P < 0.05, **P < 0.01, ns: *P* > 0.05; *n* = 3 mice, 10 dendrites from the CA1 region per group). **I** PCR expression levels of hippocampus-related synaptic indicators (*n* = 4). **J** Representative images of the western blot and **K** relative expression of glutamate receptor and synapse-related proteins (*n* = 3). Gapdh served as control. Full and uncropped western blots are detailed in the supplementary material. All the results are presented as the means ± standard deviations (SDs) with statistical significance (*P < 0.05, **P < 0.01, ns: *P* > 0.05).

We further explored whether the increase in excitatory postsynaptic transmission induced by exercise is mediated by alterations in synaptic structure. Elevated cytoplasmic Ca^2+^ concentrations increase autophagic activity [51]. Under a transmission electron microscope, we found that neurons in the CA1 region of the hippocampus of mice in the model group presented uneven chromatin, unclear nuclear membranes, swollen endoplasmic reticula and mitochondria, and a significantly reduced number of autophagosomes and autolysosomes, whereas exercise significantly ameliorated these abnormal changes (Fig. 4A, B). These results suggest that neuronal autophagy in the CA1 region of the hippocampus may mediate the neuroprotective effects of exercise in high-fat diet-fed mice. Next, the protein expression of mTOR, Beclin 1, LC3B and p62 was evaluated via Western blotting. These results further validated that exercise-induced activation of the Wnt signaling pathway increases neuronal autophagy in the CA1 region of the hippocampus to exert a neuroprotective effect (Fig. 4C, D). Golgi staining showed that a high-fat diet led to a significant reduction in the number and complexity of dendritic branches of neurons in the CA1 region of the hippocampus, but exercise effectively prevented this impairment, protecting synaptic structures from damage (Fig. 4E, F). In addition, the density of neuronal dendritic spines in the CA1 region of the hippocampus was markedly decreased in the HFD group, whereas exercise increased dendritic spine density and remodeled synaptic structure to enhance synaptic plasticity (Fig. 4G, H). To further validate that exercise significantly reverses the synaptic impairment caused by high-fat diet feeding, we used RT‒qPCR to detect changes in the expression of hippocampal synapse-related markers as well as brain-derived neurotrophic factors at the gene level (Fig. 4I). We also used western blotting to measure the expression of glutamate receptors and synapse-related proteins in the hippocampus. The results showed that high-fat diet feeding decreased the expression of glutamate receptors and synaptic proteins, which resulted in impaired synaptic structures, whereas exercise repaired synaptic damage (Fig. 4J, K). Combined with electrophysiology experiments (Fig. 3F–I), exercise remodeled synaptic structure and maintained stable synaptic plasticity. Moreover, we found that the states of microglia were also affected via PCR experiments (Fig. S5).

### Activation of Wnt5a is necessary for the exercise-induced enhancement of neuronal autophagy in the hippocampal CA1 region

**Fig. 5:**
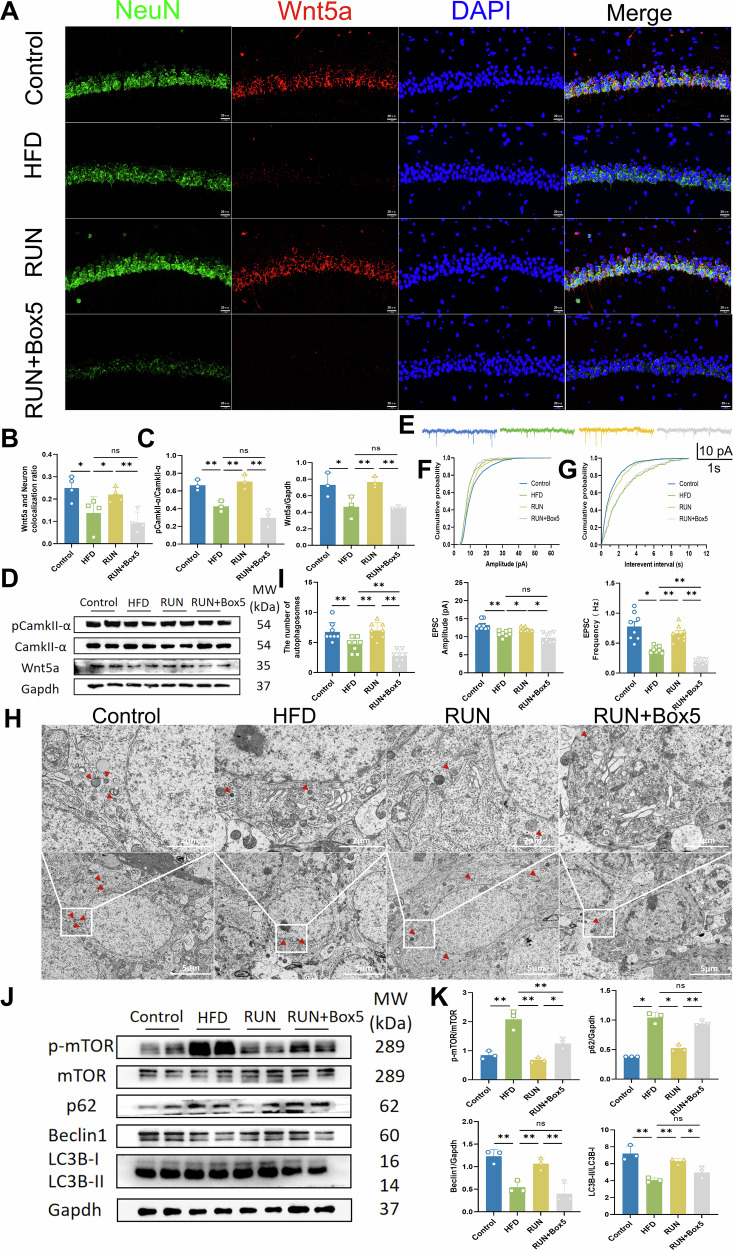
Activation of Wnt5a is necessary to increase neuronal autophagy in the hippocampal CA1 region. **A** Representative immunofluorescence image of neurons in the hippocampal CA1 region labeled with Wnt5a and NeuN (bar = 20 μm). **B** Statistical analysis of the ratio of Wnt5a to NeuN colocalization (*n* = 4). **C** Relative protein expression levels (*n* = 3) and **D** representative western blot images. Gapdh served as control. Full and uncropped western blots are detailed in the supplementary material. **E** Sample traces of excitatory postsynaptic current (EPSC). **F** Cumulative probability and comparison of the means of the EPSC amplitude (*n* = 8 from 4 mice in each group). **G** Cumulative probability and mean EPSC frequency (*n* = 8 from 4 mice in each group). **H** Ultrastructure of hippocampal neurons under a transmission electron microscope (bar = 5 μm/2 μm). Red arrows indicate autophagosomes. **I** Statistical analysis of the number of autophagosomes (*n* = 8). **J** Representative images of the western blot and **K** relative expression of p-mTOR/mTOR, p62, Beclin 1 and LC3B (*n* = 3). Gapdh served as control. Full and uncropped western blots are detailed in the supplementary material. All the results are presented as the means ± standard deviations (SDs) with statistical significance (*P < 0.05, **P < 0.01, ns: *P* > 0.05).

Box5 is an antagonist of Wnt5a that inhibits Wnt5a signaling by directly suppressing the Wnt5a-induced Ca^2+^ signaling pathway. We further explored whether Wnt5a is a key target for the antidepressant efficacy of exercise by using Box5 to inhibit Wnt5a expression in our subsequent experiments. The detailed experimental timeline is presented in Fig. S6. Inhibition of Wnt5a counteracted the ameliorative effect of exercise on depressive-like behavior in HFD-fed mice (Fig. S7). To further explore the relationship between exercise and Wnt5a-dependent neuronal autophagy, we treated mice with the Wnt5a inhibitor Box5 and fed them a high-fat diet. Immunofluorescence staining showed that Box5 counteracted the activation of Wnt5a induced by exercise (Fig. 5A, B). The Western blot results were consistent (Fig. 5C, D). According to the electrophysiological experiments, inhibition of Wnt5a resulted in significant decreases in the amplitude and frequency of excitatory postsynaptic currents in the CA1 region of the hippocampus, counteracting the enhancement of synaptic transmission by exercise (Fig. 5E–G).

However, whether Wnt5a activation is directly involved in the effect of exercise on increasing neuronal autophagy remains unclear. By transmission electron microscopy, we observed that Box5 led to the accumulation of nuclear heterochromatin, chromatin margination, swelling of the endoplasmic reticulum and mitochondria, and a significant reduction in the number of autophagosomes in the CA1 region of the hippocampus; these results were similar to those observed in the HFD group and were the opposite of those observed in the exercise group (Fig. 5H, I). Furthermore, exercise increased hippocampal Beclin 1 levels and the conversion of LC3B-I to LC3B-II in mice and decreased the expression of mTOR and the autophagy substrate p62. All these improvements were abolished by Box5 (Fig. 5J, K). In parallel, we also found that the states of microglia were also affected via PCR experiments (Fig. S8).

### Box5 suppresses the effect of exercise on synapse remodeling by inhibiting neuronal autophagy in the hippocampal CA1 region

**Fig. 6:**
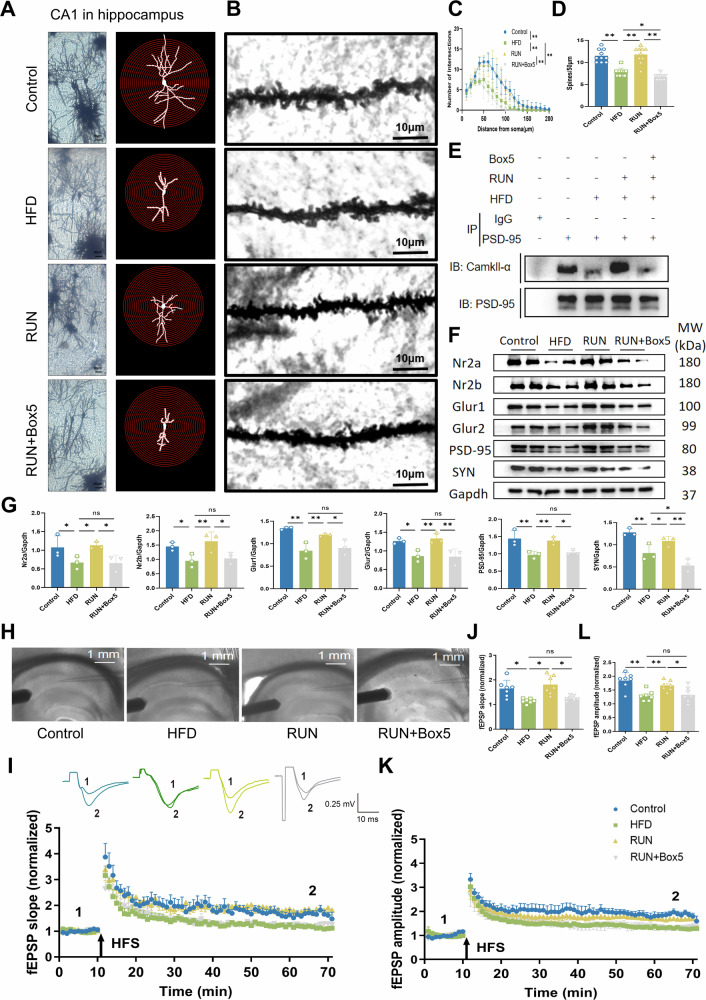
Box5 eliminates the remodeling effect of exercise on synapses by inhibiting neuronal autophagy in the hippocampal CA1 region. **A** Representative images of Golgi staining of neurons in the CA1 region of the hippocampus (bar = 20 μm). **B** Representative images of Golgi-stained dendritic spines (bar = 10 μm). **C** Fuji Sholl analysis of the dendritic complexity of neurons in the CA1 region. Statistical analysis: two-way ANOVA followed by the Tukey post hoc test (*P < 0.05, **P < 0.01, ns: *P* > 0.05; *n* = 3 mice, 2 neurons from the CA1 region per mouse). **D** Dendritic spine density. Statistical analysis: two-way ANOVA followed by Tukey’s post hoc test (*P < 0.05, **P < 0.01, ns: *P* > 0.05; *n* = 3 mice, 10 dendrites from the CA1 region per group). **E** The interaction between CamkII-α and PSD-95 was determined via coimmunoprecipitation (CO‐IP). Full and uncropped western blots are detailed in the supplementary material. **F** Representative images of the western blot and **G** relative protein expression levels are shown (*n* = 3). Gapdh served as control. Full and uncropped western blots are detailed in the supplementary material. **H** Schematic depiction of the fEPSP measurement of LTP in the Schaffer collateral pathway of the hippocampus (CA3 to CA1). **I** Representative traces and time course of fEPSP slopes during LTP recording. **J** The normalized average fEPSP slopes during the final 10 min (Control: *n* = 7 from 4 mice; HFD: *n* = 7 from 4 mice; RUN: *n* = 7 from 3 mice; RUN+Box5: *n* = 7 from 3 mice). **K** Time course of fEPSP amplitudes during LTP recording. **L** The normalized average fEPSP amplitude during the last 10 min (Control: *n* = 7 from 4 mice; HFD: *n* = 7 from 4 mice; RUN: *n* = 7 from 3 mice; RUN+Box5: *n* = 7 from 3 mice). All the results are presented as the means ± standard deviations (SDs) with statistical significance (*P < 0.05, **P < 0.01, ns: *P* > 0.05).

Normal synaptic structure is the basis for maintaining normal synaptic transmission. Golgi staining revealed more dendritic intersections, increased dendritic complexity, and a significantly increased density of dendritic spines in the RUN group than in the Control group, whereas the inhibitor Wnt5a counteracted the effects of exercise on neuronal dendritic complexity and the number of dendritic spines in the CA1 region of the hippocampus (Fig. 6A–D). We also observed by electrophysiological experiments that inhibition of Wnt5a counteracted the effect of exercise on enhancing synaptic transmission (Fig. 5E–G). These results showed that the exercise-induced enhancement of synaptic plasticity relied on synaptic remodeling through neuronal autophagy. CamkII-α and PSD-95 are postsynaptic density proteins, and their binding affinity between them affects synaptic transmission. The coimmunoprecipitation results showed that Box5 significantly attenuated the effect of exercise on increasing the binding affinity of CamkII-α for PSD-95 (Fig. 6E). Our results were further supported by western blotting. The Wnt5a inhibitor Box5 was able to counteract the reparative effects of exercise on synaptic structure in the CA1 region of the hippocampus, impairing the effect of exercise on inducing synaptic remodeling (Fig. 6F–G). To further explore the regulation of Wnt5a during exercise-induced remodeling of synapses, we completed the LTP experiment based on EPSC. The results showed that high-fat diet led to a decrease in the LTP amplitude of neurons in the hippocampal CA1 region, and that exercise was able to prevent the deficits in the induction and maintenance of LTP caused by the high-fat diet, whereas the inhibitor of Wnt5a counteracted the benefit (Fig. 6H–L).

## Discussion

In recent years, the consumption of high-energy foods, especially high-fat foods, has become increasingly common among adults, leading to a substantial increase in demand for such foods [1]. Although dietary fat is an important source of the long-chain polyunsaturated fatty acids (LCPUFA) necessary to maintain early central nervous system development and cognitive function, long-term consumption of a high-fat diet is associated with a high incidence of metabolic diseases, cognitive impairment, and mental disorders [52, 53]. However, the underlying mechanisms of HFD-induced neurobehavioral changes remain incompletely understood. Consistent with the findings of previous studies [2, 54, 55], the findings of the present study indicated that long-term HFD consumption not only negatively affects the body’s metabolism but also leads to depression-like behavior and hippocampal neuronal damage, accompanied by a certain degree of neuroinflammation. The effects of a high-fat diet on the brain are widespread. A long-term high-fat diet leads to a chronic inflammatory state, and microglia are activated by inflammatory factors followed by further secretion of inflammatory factors, resulting in a vicious cycle [56, 57]. In this vicious cycle, synapses are abnormally pruned, neurons are structurally damaged, and synaptic plasticity is impaired, further exacerbating depressive-like behaviors [58, 59]. Previous research has focused mostly on the harm caused by long-term HFD consumption on brain health, whereas limited attention has been given to nonpharmacological interventions such as exercise. Our study suggested that tailored exercise interventions may serve as a preventive and therapeutic strategy for unhealthy high-fat diet consumption.

Regular exercise has various health benefits and is often recommended to combat obesity [60, 61]. Recently, exercise has been shown to have beneficial effects on brain function and is recommended as a nonpharmacological therapy for depression [32, 33]. The pathogenesis of depression is complex; in addition to neurons, astrocytes and microglia are also involved, which ultimately reduces neuroplasticity [62]. In this study, we focused on neuronal synapses. More and more studies show that astrocytic leaflets establish contacts with synapses and maintain synaptic connectivity [63]. These astrocytic leaflets and branches interact with synapses, dendrites, and axons, playing crucial roles in the morphological organization and functional activity of the active milieu [64]. In future studies, we will explore how exercise affects the neuronal synaptic function via astrocytes. The lifelong plasticity of the hippocampus contributes to maintaining normal emotional functions, but this brain region is also highly susceptible to the consumption of an unhealthy diet and external stressors [65, 66]. Previous studies have shown that the different effects of a high-fat diet on LTP are related to the duration of exposure to the high-fat diet, the fat composition, the genetic background of the model animals, and their age [3, 5, 67, 68]. In the present study, long-term consumption of high-fat diet markedly decreased the density of neuronal dendritic spines, amplitude of LTP and EPSC in the CA1 region of the hippocampus, whereas exercise could remodel synaptic structure and maintain stable synaptic plasticity. This finding is consistent with previous research [3, 67], and we further demonstrated the preventive and ameliorative effects of exercise training on these abnormalities.

To explore the mechanisms by which exercise alleviates the effects of HFD consumption, we conducted transcriptome sequencing of hippocampal tissue, suggesting that Wnt5a played a key role in this. Wnt5a is expressed not only in neurons but also in glial cells and may serve as a pathway for communication between neurons and glial cells [69]. Combined with the results of correlation analysis and immunofluorescence colocalization experiments, these findings suggest that Wnt5a in neurons may be a key target of exercise intervention for treating HFD-induced depressive-like behaviors. Previous research involving neuron-specific deletion of Wnt5a in mice showed that Wnt5a is involved in regulating axon and dendrite growth, as well as synapse formation, which are essential for maintaining long-term dendritic stability in the adult hippocampus [50]. Although previous research has focused primarily on the impact of Wnt5a loss on hippocampal synaptic plasticity and spatial learning and memory in adult mice [50, 69], our study aimed to explore its effects on emotional disorders. Wnt5a is involved in the noncanonical Wnt signaling pathway, and the binding of Wnt5a to its receptor Frizzled triggers a cascade of reactions, leading to increased Ca^2+^ release, activation of calcium/calmodulin-dependent kinase II (CamkII), and alterations in neuronal development and synapse formation [50]. In our study, exercise increased synaptic plasticity to improve synaptic transmission efficiency and restored Wnt5a/Ca^2+^/CamkII signaling inhibited by HFD, but the specific mechanisms involved remain unclear. Therefore, we hypothesize that regular exercise, through the regulation of the Wnt5a/CamkII pathway, activates hippocampal neuron autophagy and enhances synaptic plasticity, thus preventing or alleviating depressive-like behavior induced by HFD consumption.

In the mature central nervous system, autophagy plays a role in plasticity through its effects on axons, dendritic spines, and synaptic assembly processes, and defects in autophagy are thought to contribute to human diseases such as depression, bipolar disorder, and schizophrenia [27]. Physical exercise regulates autophagy in the nervous system by regulating autophagy-related factors, transcription of important autophagy genes, autophagic flux and accumulation of autophagosomes and plays crucial roles in neuroprotection [37]. Therefore, physical exercise is expected to prevent neuronal damage or promote neuronal functional recovery by regulating autophagy in a variety of neurological diseases [70].

Moreover, exercise was found to influence mTOR signaling in high-fat diet-fed mice, which is consistent with the findings of our previous studies [40, 42, 71]. However, many scholars have focused only on whether mTOR activation may increase BDNF and synaptic protein levels to promote synaptic remodeling [71, 72]. Notably, under physiological conditions, mTOR is involved in various pathways, and its activation can inhibit autophagy. Interestingly, recent research has revealed that CamkII signaling in the hippocampus may negatively regulate mTOR signaling, suggesting that mTOR activity might be suppressed by CamkII phosphorylation, thus restoring normal autophagy levels [72]. Based on these findings, we hypothesize that regular exercise activates hippocampal neuronal autophagy to enhance synaptic plasticity through the regulation of the Wnt5a/CamkII pathway, thereby preventing or alleviating depression-like behavior induced by a high-fat diet. The experimental results indicated that a high-fat diet significantly reduced dendritic branching complexity and induced autophagic impairment in hippocampal CA1 neurons, which is consistent with the findings of previous studies [20, 22]. To further investigate whether exercise-induced neuronal autophagy depends on Wnt5a, we conducted experiments using the Wnt5a antagonist Box5. Consistent with our hypothesis, inhibiting Wnt5a/Ca^2+^ signaling with Box5 prevented the exercise-mediated alleviation of depression-like behavior induced by high-fat diet feeding and did not increase hippocampal neuron autophagy, synaptic signal transmission, or synaptic structural remodeling. Therefore, in our study, activation of Wnt5a was necessary for exercise to enhance hippocampal CA1 neuron autophagy.

In conclusion, the current study reveals a novel mechanism that connects HFD consumption, Wnt5a/CamkII signaling, neuronal autophagy, and exercise therapy. This mechanism provides crucial insights into the prevention and treatment of diet-induced neurological disorders.

## Materials and methods

### Animals and the model

Six-week-old male C57BL/6J mice weighing 19 ± 1 g were purchased from Beijing Vital River Laboratory Animal Technology Corporation (Beijing, China). The mice were housed under a 12-h light–dark cycle in a controlled environment (22 ± 1 °C) and allowed free access to food and water. All the experimental procedures were conducted in accordance with the guidelines for the management of experimental animals and approved by the Animal Ethics Committee of Jinan University (IACUC-20221206-02).

Experiment 1: Thirty-six mice were randomly divided into Control, HFD, and RUN groups using the random number table method (*n* = 12). The Control group received standard chow, whereas the other groups received a HFD (60% kcal from fat) [43] for 8 weeks (Supplementary Table 1). The mice in the RUN group exercised on treadmill equipment (Zhongshi Tech., China) at a fixed time (4:00 pm–5:00 pm) for 1 h every day at a speed of 10 m/min [40–42, 44–47].

Experiment 2: Forty-eight mice were randomly divided into Control, HFD, RUN, and RUN+Box5 groups using the random number table method (*n* = 12). The mice in the RUN+Box5 group were subjected to exercise and treated with Box5 (1 mg/kg; P1216; Selleck, Texas, USA), a Wnt5a inhibitor [48].

The specific experimental methods mentioned in this article are described in the Supplementary Materials. For more details, see the Supplementary Materials.

### Statistical analysis

SPSS 26.0 (version 26.0.1; Armonk, NY, USA) was used for analysis, and all the results are presented as the means ± standard deviations (SDs). One-way ANOVA was used to compare among multiple groups, and repeated-measures ANOVA was used for within-group comparisons. For normally distributed data, differences between groups were analyzed via Tukey’s post hoc test; otherwise, Tamhane’s *T*^2^ test was used. For experiments with two independent variables, two-way ANOVA followed by the Bonferroni post hoc correction was performed. *P* < 0.05 indicated statistical significance. All the statistical data were plotted via GraphPad Prism 9.0 (GraphPad Software, La Jolla, CA, USA).

## Supplementary information

Supplementary materials

Original Western Blots

## Data Availability

The RNA-seq data reported in this study have been deposited in the Sequence Read Archive (SRA), at the National Center for Biotechnology Information (BioProject Accession: PRJNA 1123399). The additional data used to support the findings are available from the corresponding author upon request.
