# Supplementary material for: Regular exercise ameliorates high-fat diet-induced depressive-like behaviors by activating hippocampal neuronal autophagy and enhancing synaptic plasticity
Source: Cell Death Dis. 2024 Oct 10;15(10):737. doi: 10.1038/s41419-024-07132-4 (PMC11467387; doi:10.1038/s41419-024-07132-4)
Supplement: Supplementary file 1 — Supplementary materials [file 41419_2024_7132_MOESM1_ESM.docx]

**Supplementary materials**

**Original article**

**Regular exercise ameliorates High-Fat Diet-induced depressive-like behaviors by activating hippocampal neuronal autophagy and enhancing synaptic plasticity**

Jialin Wu^1,3^, Huachong Xu^1,2,3,^*, Shiqi Wang^1^, Huandi Weng^1,2^, Zhihua Luo^2^, Guosen Ou^1^, Yaokang Chen^1^, Lu Xu^1^, Kwok-Fai So^2^, Li Deng^1,^*, Li Zhang^2,^*, Xiaoyin Chen^1,^*

^1^ School of Traditional Chinese Medicine, Jinan University, Guangzhou 510632, China

^2^ Key Laboratory of Central CNS Regeneration (Ministry of Education), Guangdong-Hong Kong-Macau Institute of CNS Regeneration, Jinan University, Guangzhou 510632, China

^3^ These authors contributed equally: Jialin Wu, Huachong Xu.

* Corresponding: xuhuachong@jnu.edu.cn (H. Xu); dengli@jnu.edu.cn (L. Deng); zhangli@jnu.edu.cn (L. Zhang); tchenxiaoyin@jnu.edu.cn (X. Chen).

**Methods**

**Behavioral assessment**

Depressive-like behavior was assessed via the following four behavioral tests, all of which were conducted in a quiet, undisturbed environment; EthoVision XT software (Noldus, Wageningen, Netherlands) was used to analyze the behavior of the mice.

1. Open Field Test (OFT)

Each mouse was gently placed in the center of a black plastic square (50 cmx50 cmx40 cm) and allowed to explore freely for 8 min, and the activities of the mice were recorded with a camera.

1. Tail Suspension Test (TST)

Medical tape was tied around the tail of each mouse was tied, and the mice were then suspended from a hook at a height of 50 cm. The mice were allowed to move freely for 6 min, and the total immobilization time was recorded.

1. Forced Swimming Test (FST)

A clear glass round bucket was filled to a height of 30 cm with water, and the temperature of the water was maintained at 21–23°C. Each mouse was placed in a separate bucket and allowed to swim for 5 min, and the total immobilization time was recorded.

1. Sucrose Preference Test (SPT)

Each mouse was individually housed in a cage and given two bottles filled with 2% sucrose, one of which was replaced with a water-filled bottle after 24 h. The position of the water bottles was switched every 24 h to prevent the mice from exhibiting a positional preference. After training for 72 h, the sucrose and water consumption of each mouse at 24 h was recorded, and the sucrose preference was calculated (sucrose preference = volume (sucrose solution) / (volume (sucrose solution) + volume (water) ) / 100).

**Body weight and liver index**

The weights of the mice were recorded daily. After the mice were anesthetized with isoflurane (R510-22-10, REWARD, Shenzhen, China), their body size was photographed, and the abdomens of the mice were dissected and the abdominal fat of the mice was photographed under sterile conditions. Afterwards, the liver and epididymal fat of the mice were removed and weighed. The formula for calculating the liver index was as follows: Liver index = liver weight (g)/body weight (g)*100%.

**Biochemical parameters analysis**

After blood was collected through the retro-orbital sinus, the mice were sacrificed by cervical dislocation. The serum was collected by centrifugation at 3000 rpm for 10 min at room temperature. The serum TC (Cat#: S03042), TG (Cat#: S03027), LDL-C (Cat#: S03029) and HDL-C (Cat#: S03025) levels were assayed via kits (Rayto, Shenzhen, China).

**ELISA**

ELISA kits (Jiangsu Meimian Industrial Co., Ltd., Jiangsu, China) were used to measure the levels of IL-6 (Cat#: MM-0163M1), TNF-α (Cat#: MM-0132M1), IL-4 (Cat#: MM-0165M1), and IL-10 (Cat#: MM-0176M1) in mouse hippocampal tissues following the manufacturer’s protocol.

**Nissl staining**

The brains were fixed with 4% paraformaldehyde (G1101; Servicebio, Wuhan, China), embedded in paraffin (G1101; Servicebio, Wuhan, China), and deparaffinized. Subsequently, 4-μm-thick hippocampal slices were cut transversely using a vibrating slicer (Leica, Germany). The brain slices were stained with reagents from a Nissl staining kit (Servicebio, Wuhan, China), and a microscope (ZEISS, Germany) was used to observe the arrangement and number of neurons in the CA1 region of the hippocampus.

**RNA sequencing**

Total RNA was isolated and purified using TRIzol reagent (Invitrogen, Carlsbad, CA, USA) following the manufacturer's protocol. The RNA concentration and purity of each sample were quantified using a NanoDrop ND-1000 spectrophotometer (NanoDrop, Wilmington, DE, USA). RNA sequencing was performed on an Illumina NovaSeq™ 6000 platform (LC-Bio Technology Co., Ltd., Hangzhou, China) following the vendor’s recommended protocol.

**Immunofluorescence staining**

Slices were prepared as described above. The slices were blocked with goat serum (16210064; Gibco, New York, USA) and incubated with primary antibodies against Wnt5a (1:200; PTG, Wuhan, China), NeuN (1:100; PTG, Wuhan, China), GFAP (1:800; PTG, Wuhan, China) and IBA-1 (1:500; PTG, Wuhan, China) overnight at 4°C, followed by incubation with secondary antibodies at 37°C for 1 h. The tissues were incubated with TSA-570 (PN1006; Wuhan, China) for 30 min at 37°C, the cell nuclei were stained with DAPI, and the slices were mounted with anti-fluorescence quenching reagent (SouthernBiotech, Beijing, China). Images were captured using a confocal microscope (LSM 800; Zeiss, Oberkochen, Germany), and the fluorescence intensity was analyzed using ImageJ (National Institutes of Health, Bethesda, MD, USA).

**Acute slice preparation for electrophysiology**

Under isoflurane anesthesia, the brain was rapidly removed, and hippocampal coronal slices were obtained with a vibratome (Leica Microsystems, Wetzlar, Germany). The slices were incubated in ice-cold, oxygenated (95% O2 and 5% CO2) ACSF. Slices were recovered at 37°C for 60 min in a chamber filled with oxygenated artificial cerebrospinal fluid (ACSF) and were then stored at room temperature until needed.

**EPSC recordings**

Filamentary borosilicate glass capillaries were pulled into recording electrodes with a resistance in the range of 4-7 MΩ. Excitatory postsynaptic currents (EPSCs) in slices of the hippocampal CA1 region (250 µm) were recorded in the presence of 1 μM TTX and 20 μM bicuculline, and the electrodes were filled with K+-based peptide solution. Traces were collected by a MultiClamp 700B amplifier (Molecular Devices, USA), and the data were analyzed via Clampfit 10.0 (Molecular Devices, USA).

**LTP recordings**

Hippocampal slices (250 µm) were transferred to a recording chamber and carbogen-saturated ACSF was perfused constantly at room temperature. A concentric circular stimulating electrode was placed in the Schaffer collateral to deliver electrical stimuli. A glass microelectrode filled with ACSF was positioned in the CA1 stratum radiatum to record field excitatory postsynaptic potentials (fEPSPs). fEPSPs were evoked with an intensity (1 ms) that elicited ~50% of the maximum amplitude at 0.05 Hz. LTP was induced with an HFS (high-frequency stimulation) protocol comprising two consecutive 1-s trains of 100-Hz stimuli, with a 20-s interval between trains. Potentiation was calculated as the percent increase of the average fEPSP slopes during

the last 10 min normalized to the average of the baseline slopes. The signals were acquired with a MultiClamp 700B amplifier (Molecular Devices, USA). The data were analyzed with Clampfit 10.0 (Molecular Devices, USA).

**Transmission electron microscope**

Hippocampal tissues were fixed with an electron microscope fixative (Servicebio, Wuhan, China), transferred to 1% osmium acid, fixed for 2 h, dehydrated, and embedded in osmosis embedding agent for 3 h. The resin blocks were sliced into 60-80 μm sections and stained with 2% uranyl acetate-saturated alcohol solution. Finally, the slices were observed, and images were acquired under a transmission electron microscope (Hitachi, Tokyo, Japan).

**Golgi staining**

After fixation for 24 hours using Golgi staining fixative (G1069-15ML; Servicebio, Wuhan, China), the brains were submerged in Golgi staining solution (G1069-1; Servicebio, Wuhan, China). Coronal slices of the hippocampus with a thickness of 60 μm were prepared with a vibratome, and the neuronal structure was observed via a confocal microscope. The morphology of each neuron was evaluated by Fiji software (version, Inc.). Sholl rings were drawn from the center of the cell body to the end of the dendrites at intervals of 10 μm, and the number of intersections between the Sholl rings and the neuronal dendrites was counted. The morphology of the dendritic spines was observed under a microscope with a 63X oil lens, and the number of dendritic spines along each 50 μm segment was counted.

### **Western blotting**

Hippocampal tissues were lysed using RIPA lysis buffer (Beyotime Biotechnology, Shanghai, China), and a BCA protein concentration measurement kit (Solebo, Beijing, China) was used to determine the protein concentration. After electrophoresis, the PVDF membranes (Millipore, Massachusetts, USA) were incubated overnight at 4°C with primary antibody, followed by incubation with the corresponding secondary antibody (Supplementary Table 2). The protein bands were imaged with a ChemiDoc imaging system (1708280; Bio-Rad, California, USA), and the gray values of the protein bands were analyzed via ImageJ.

**Coimmunoprecipitation**

Hippocampal tissue was lysed using RIPA lysis buffer and incubated with an anti-PSD95 antibody overnight at 4°C; then, the proteins were mixed with Protein A agarose beads (sc-2003, Santa Cruz, Dallas, Texas, USA). After sufficient immunoprecipitation, the supernatant was centrifuged and analyzed by Western blotting.

**Quantitative polymerase chain reaction**

RNA was extracted from the hippocampus with TRIzol (9109; Takara, Shiga, Japan). cDNA was synthesized following the instructions of the PrimeScript™ RT Reagent Kit with gDNA Eraser (RR047; Takara, Shiga, Japan), and fluorescence quantitative PCR was subsequently performed with TB Green® Premix Ex Taq™ (RR820; Takara, Shiga, Japan) in a CFX96 Real-Time device (CFX96 Touch, Bio-Rad, California, USA). The primer sequences are shown in Supplementary Table 3.

**Supplementary Table 1. The high-fat diet formula.**

| **Product #D12492** | **gm%** | **kcal%** |
| --- | --- | --- |
| Protein | 26.2 | 20 |
| Carbohydrate | 26.3 | 20 |
| Fat | 34.9 | 60 |
| **Total** |  | 100 |
| **kcal/gm** | 5.24 |  |
|  |  |  |
| **Ingredient** | **gm** | **kcal** |
| Casein, 30 Mesh | 200 | 800 |
| L-Cystine | 3 | 12 |
|  |  |  |
| Corn Starch | 0 | 0 |
| Maltodextrin 10 | 125 | 500 |
| Sucrose | 68.8 | 275.2 |
|  |  |  |
| Cellulose, BW200 | 50 | 0 |
|  |  |  |
| Soybean Oil | 25 | 225 |
| Lard* | 245 | 2205 |
|  |  |  |
| Mineral Mix S10026 | 10 | 0 |
| DiCalcium Phosphate | 13 | 0 |
| Calcium Carbonate | 5.5 | 0 |
| Potassium Citrate, 1 H2O | 16.5 | 0 |
|  |  |  |
| Vitamin Mix V10001 | 10 | 40 |
| Choline Bitartrate | 2 | 0 |
|  |  |  |
| FD&C Blue Dye #1 | 0.05 | 0 |
|  |  |  |
| **Total** | **773.85** | **4057** |

**Supplementary Table 2. Table of antibodies used.**

| **Antibody** | **RRID:** | **Supplier** | **Concentration** |
| --- | --- | --- | --- |
| Gapdh (primary) | 5174 | CST (Danvers, MA. USA) | 1:1000 |
| Wnt5a (primary) | bs-1948R | Bioss (Beijing, China) | 1:1000 |
| p-CaMKII-α (primary) | ab171095 | Abcam (Cambridge, UK) | 1:1000 |
| CamkII-α (primary) | 50049 | CST (Danvers, MA. USA) | 1:1000 |
| CamkII-α (primary) | sc-13141 | Santa (Dallas, Texas, USA) | 1:50 |
| p-mTOR (primary) | 5536 | CST (Danvers, MA. USA) | 1:1000 |
| mTOR (primary) | 2983 | CST (Danvers, MA. USA) | 1:1000 |
| Beclin 1 (primary) | 3495 | CST (Danvers, MA. USA) | 1:1000 |
| p62 (primary) | 23214 | CST (Danvers, MA. USA) | 1:1000 |
| LC3B (primary) | 2775 | CST (Danvers, MA. USA) | 1:1000 |
| PSD95 (primary) | ab238135 | Abcam (Cambridge, UK) | 1:1000 |
| SYN (primary) | 36406 | CST (Danvers, MA. USA) | 1:1000 |
| Nr2a (primary) | AB1555P | Millipore (MA, USA) | 1:1000 |
| Nr2b (primary) | AB1557P | Millipore (MA, USA) | 1:1000 |
| Glur1 (primary) | ab31232 | Abcam (Cambridge, UK) | 1:1000 |
| Glur2 (primary) | ab20673 | Abcam (Cambridge, UK) | 1:1000 |
| Goat anti-Mouse IgG-HRP Antibody (secondary) | abs20039 | Absin (Shanghai, China) | 1:5000 |
| Goat anti-Rabbit IgG-HRP Antibody (secondary) | abs20040 | Absin (Shanghai, China) | 1:5000 |

**Supplementary Table 3. Table of primers used.**

| **Gene Name** | **Primers (5’-3’)** | |
| --- | --- | --- |
| Gapdh | Forward: | AGAAGGTGGTGAAGCAGGCATC |
|  | Reverse: | CGAAGGTGGAAGAGTGGGAGTTG |
| Wnt5a | Forward: | CTGCGGAGACAACATCGACTA |
|  | Reverse: | CGTGGATTCGTTCCCTTTCTCTA |
| Camkll-α | Forward: | ACAGAGCAGCTGATCGAAGC |
|  | Reverse: | AGGTGGATGTGAGGGTTCAG |
| Zfp740 | Forward: | CCATCTCGAACGCCACAAG |
|  | Reverse: | TGTCTGAGTAATCGGTCTGTCC |
| Septin2 | Forward: | ATTGTGCCTGTCATTGCGAAA |
|  | Reverse: | CCTTGAGGAGTCTAGTCTGCT |
| Scn3b | Forward: | ATGTGTCCAGGGAGTTTGAGT |
|  | Reverse: | TTCGGCCTTAGAGACCTTTCT |
| PSD-95 | Forward: | AGGGGAGGAACAAAACTCCA |
|  | Reverse: | AGGGGGAGAATTGGCCTGG |
| SYN | Forward: | TGGAGTGTGCCAACAAGAC |
|  | Reverse: | AGCCACGGTGACAAAGAA |
| BDNF | Forward: | GTAAAGCCAACCCTGTGTCG |
|  | Reverse: | TCCGCTCCAAAATCTGACTC |
| Iba1 | Forward: | GGATTTGCAGGGAGGAAAAG |
|  | Reverse: | TGGGATCATCGAGGAATTG |
| CD11b | Forward: | GAGGCCCCCAGGACTTTAAC |
|  | Reverse: | CTTCTTGGTGAGCGGGTTCT |
| CD68 | Forward: | TGTCTGATCTTGCTAGGACCG |
|  | Reverse: | GAGAGTAACGGCCTTTTTGTGA |
| CD86 | Forward: | ACGATGGACCCCAGATGCACCA |
|  | Reverse: | GCGTCTCCACGGAAACAGCA |
| NOX2 | Forward: | GGGAACTGGGCTGTGAATGA |
|  | Reverse: | CAGTGCTGACCCAAGGAGTT |
| iNOS | Forward: | GCTATGGCCGCTTTGATGTG |
|  | Reverse: | TCGAACTCCAATCTCGGTGC |
| CD206 | Forward: | TCAGCTATTGGACGCGAGGCA |
|  | Reverse: | TCCGGGTTGCAAGTTGCCGT |
| CD163 | Forward: | CTCTGAATGACCCCCGAGGA |
|  | Reverse: | CACGGCACTCTTGGTTTGTG |
| Ym1 | Forward: | ACCCCTGCCTGTGTACTCACCT |
|  | Reverse: | CACTGAACGGGGCAGGTCCAAA |
| Arg1 | Forward: | TTAGGCCAAGGTGCTTGCTGCC |
|  | Reverse: | TACCATGGCCCTGAGGAGGTTC |


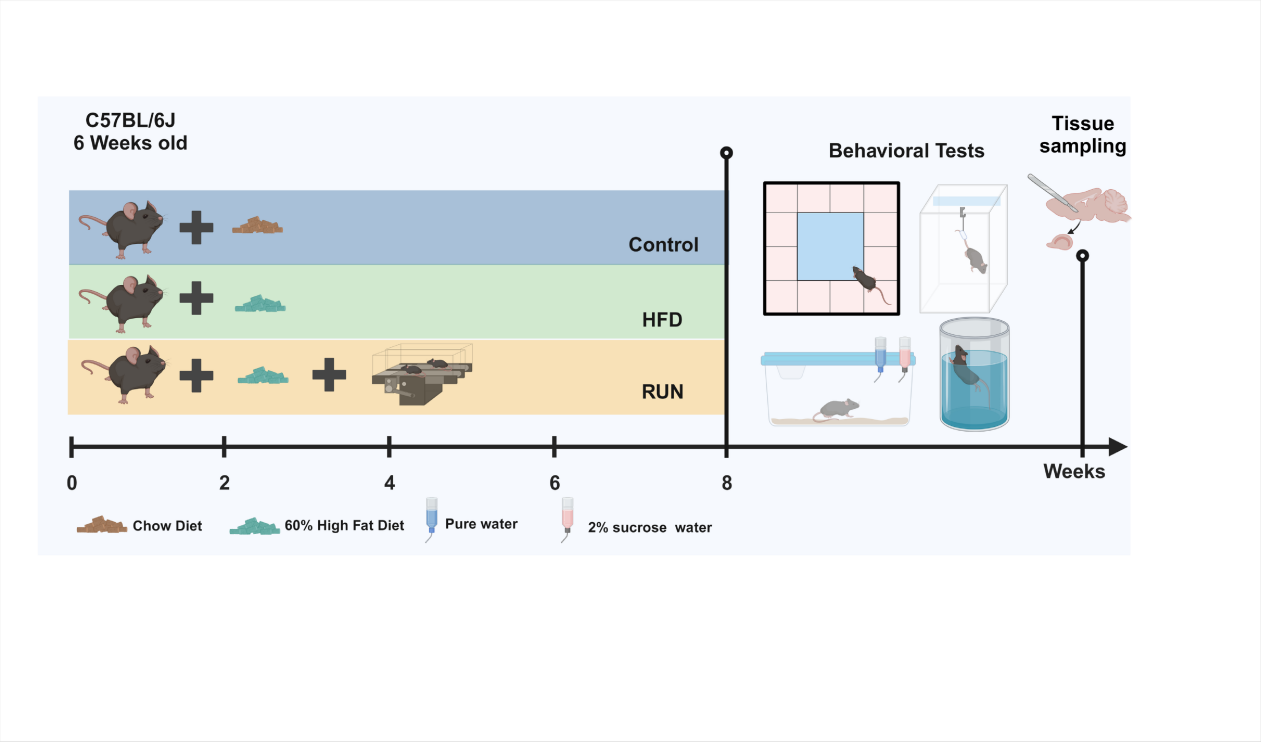


**Fig. S1. Study timeline 1.** Control: The group given standard chow. HFD: The group given a high-fat diet (60% kcal from fat). RUN: The group given a high-fat diet (60% kcal from fat) and exercised on treadmill equipment at the same time. (Created with BioRender.com)


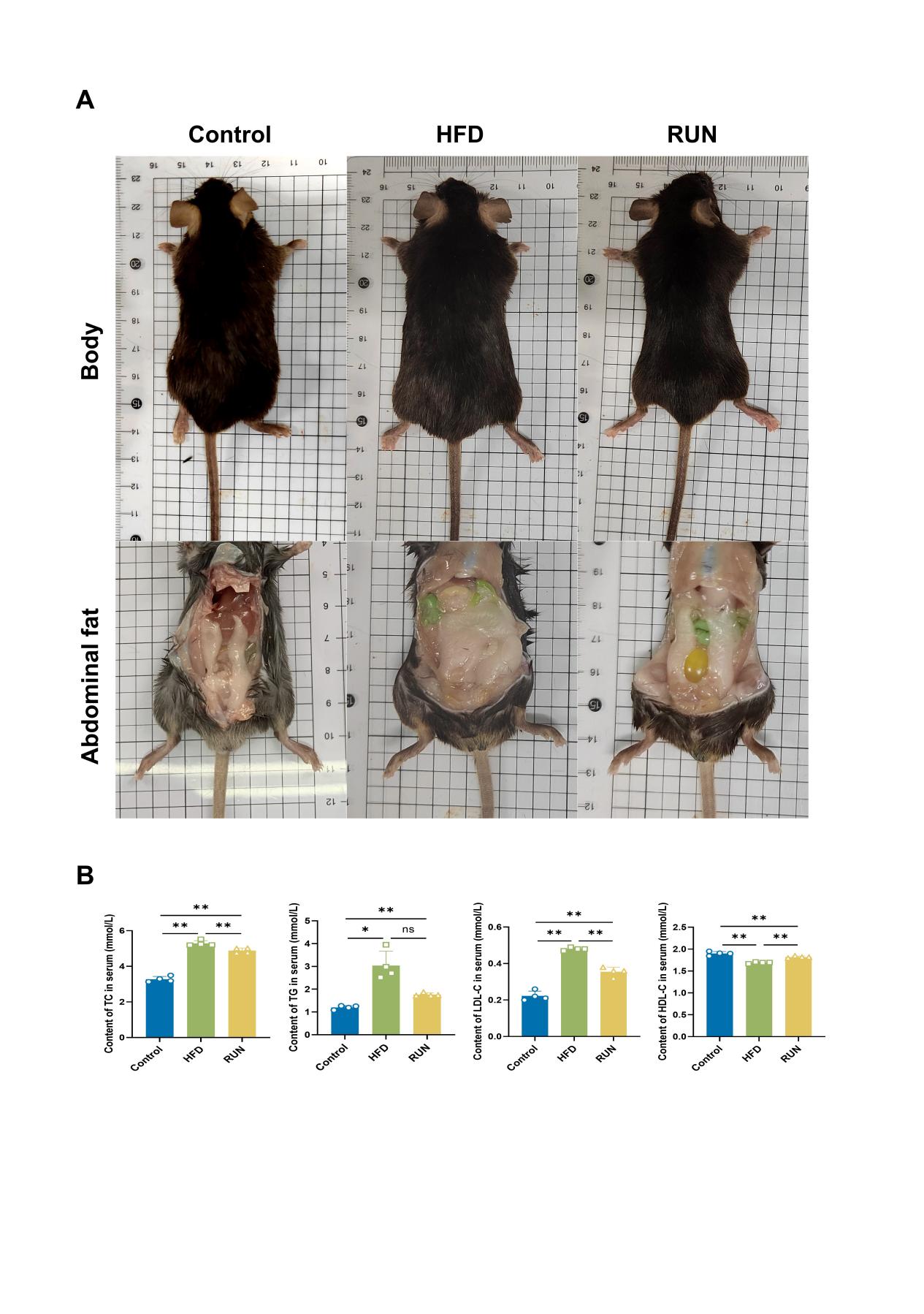


**Fig. S2. Mouse body size, abdominal fat and lipid levels.** (A) Representative images of mouse body size and abdominal fat. (B) Serum lipid levels (n = 4). All the results are presented as the means ± standard deviations (SDs) with statistical significance (**P* < 0.05, ***P* < 0.01, ns: *P* > 0.05).

**
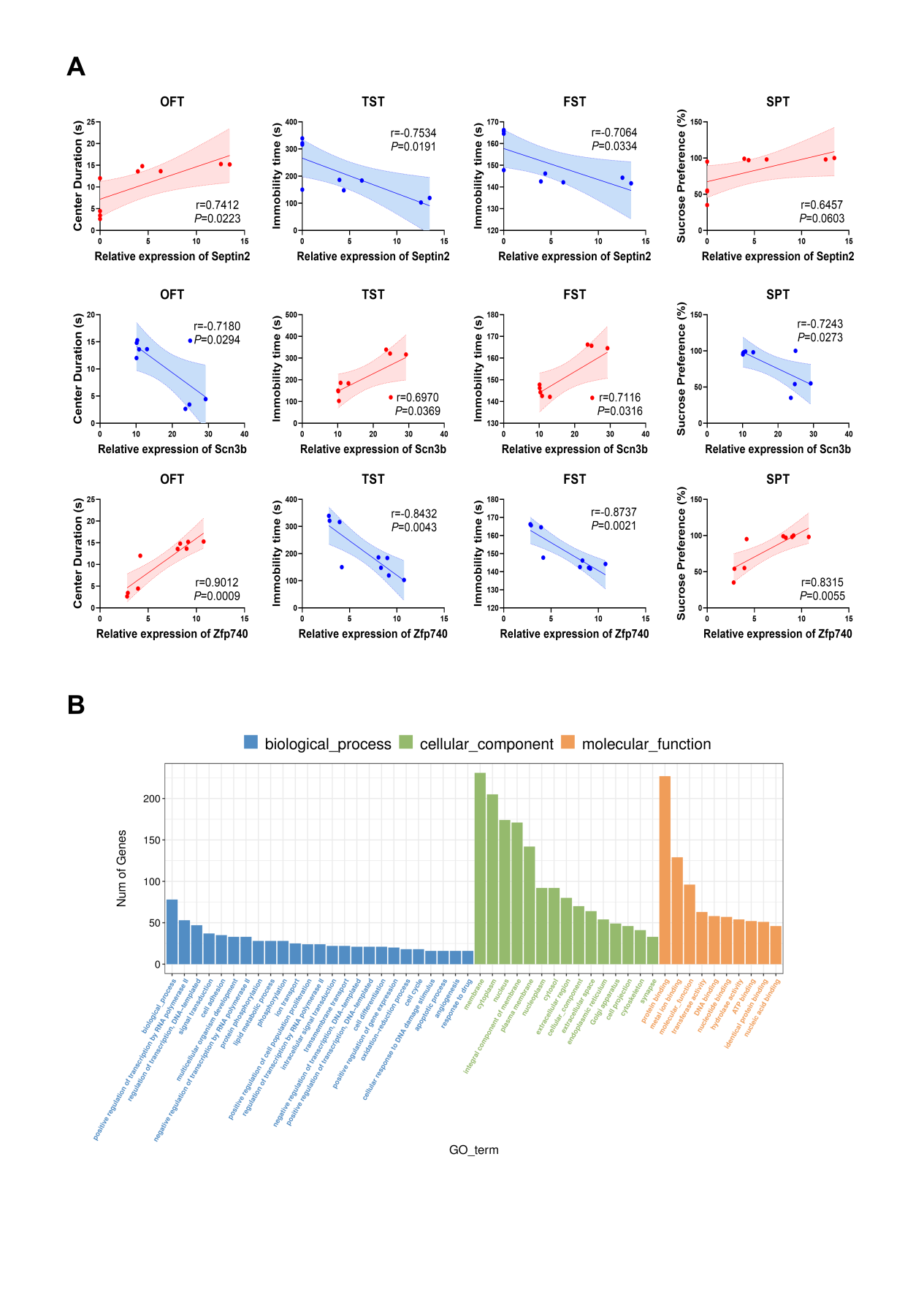
**

**Fig. S3. Correlation analysis between genes and behavioral tests.** (A) Correlation analysis of Septin2, Scn3b and Zfp740 with behavioral tests respectively (n = 3). (B) GO enrichment analysis.


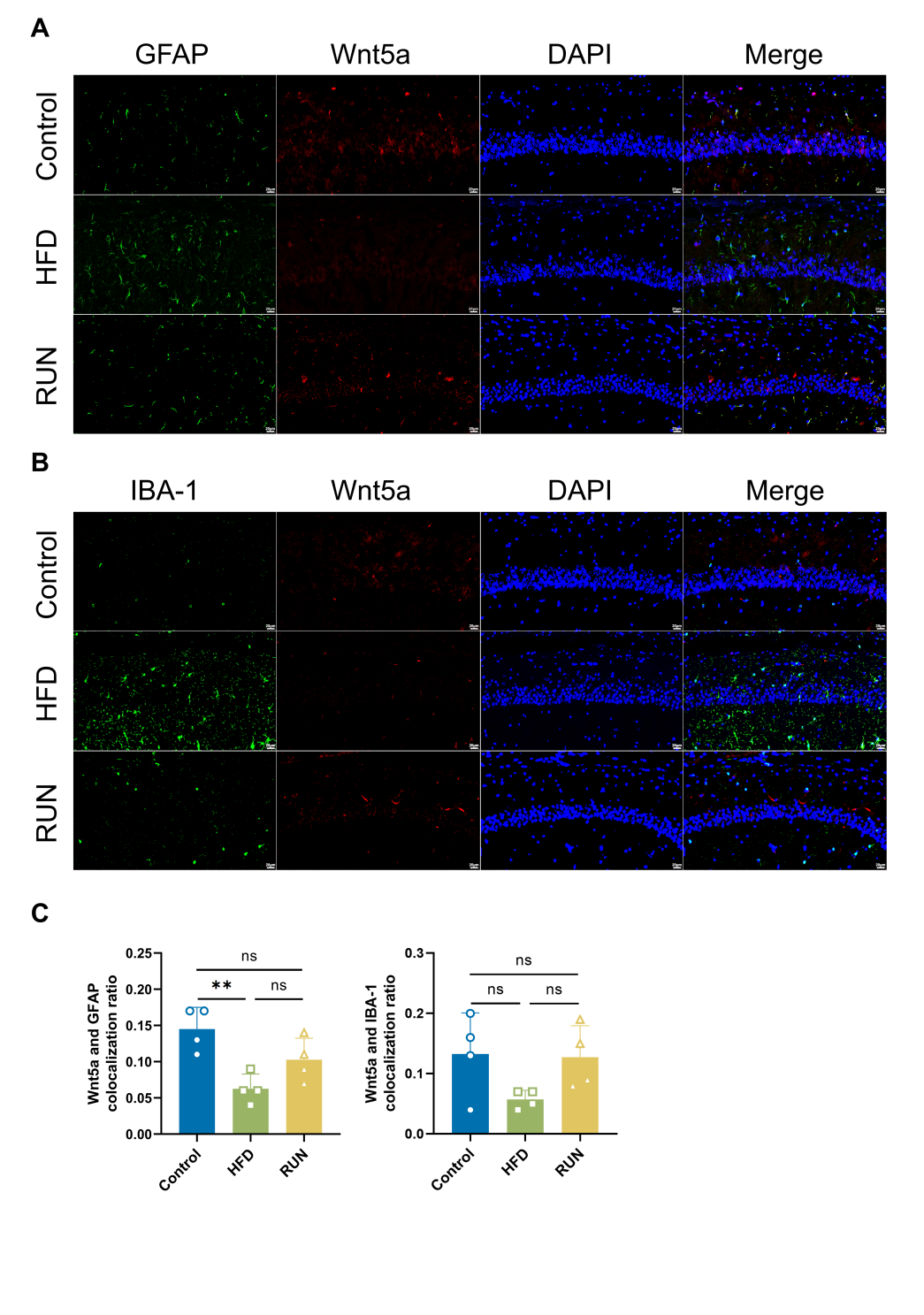


**Fig. S4. Changes in astrocytes and microglia in the CA1 region of the mouse hippocampus.** (A) Representative immunofluorescence image of astrocytes in the hippocampal CA1 region labeled with Wnt5a and GFAP (bar = 20 μm). (B) Representative immunofluorescence image of microglia in the hippocampal CA1 region labeled with Wnt5a and IBA-1 (bar = 20 μm). (C) Statistical analysis of the ratio of Wnt5a to GFAP, IBA1 colocalization (n = 4). All the results are presented as the means ± standard deviations (SDs) with statistical significance (**P* < 0.05, ***P* < 0.01, ns: *P* > 0.05).

**
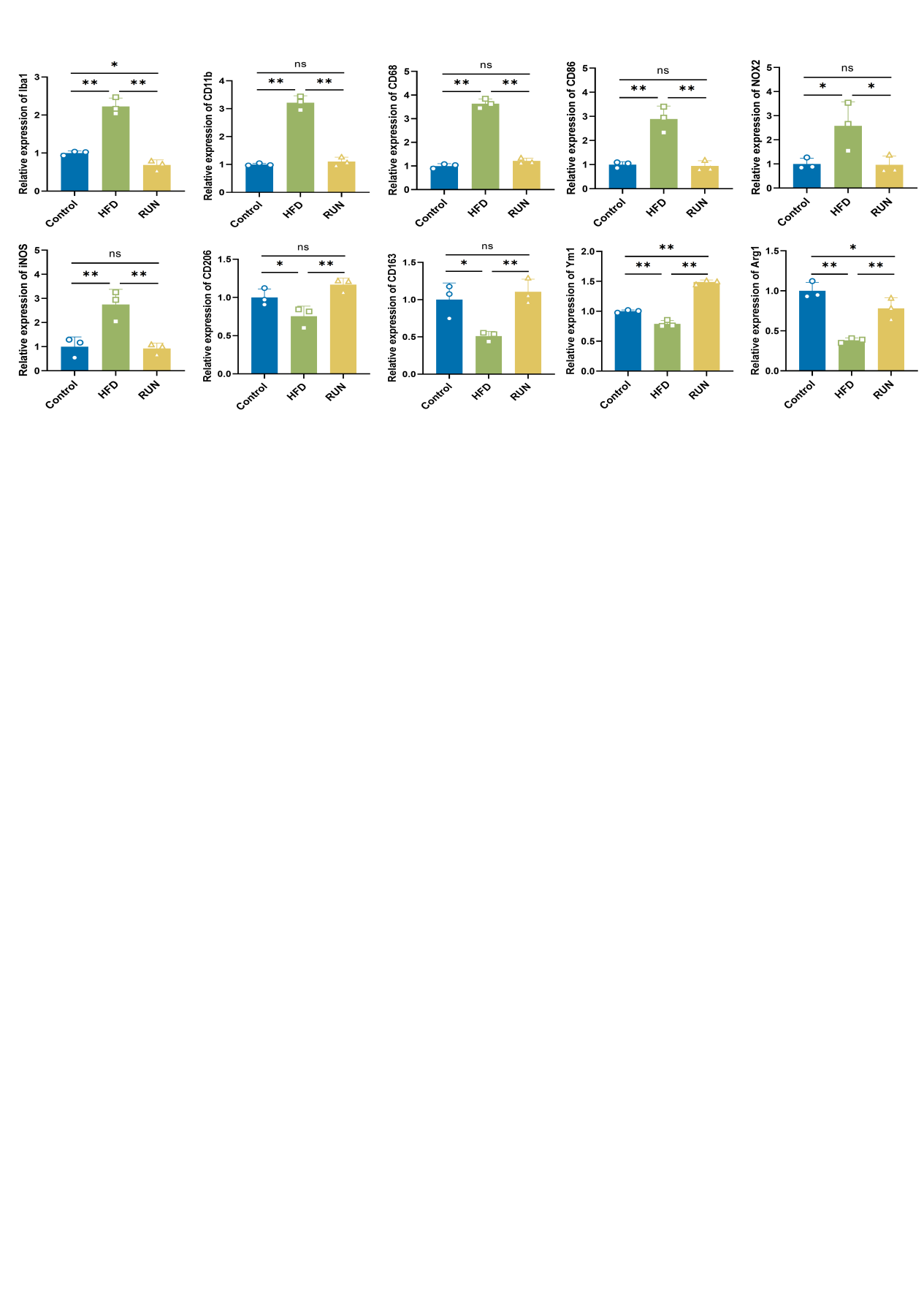
**

**Fig. S5. Relative expression of microglial cell surface markers (n = 3).** All the results are presented as the means ± standard deviations (SDs) with statistical significance (**P* < 0.05, ***P* < 0.01, ns: *P* > 0.05).


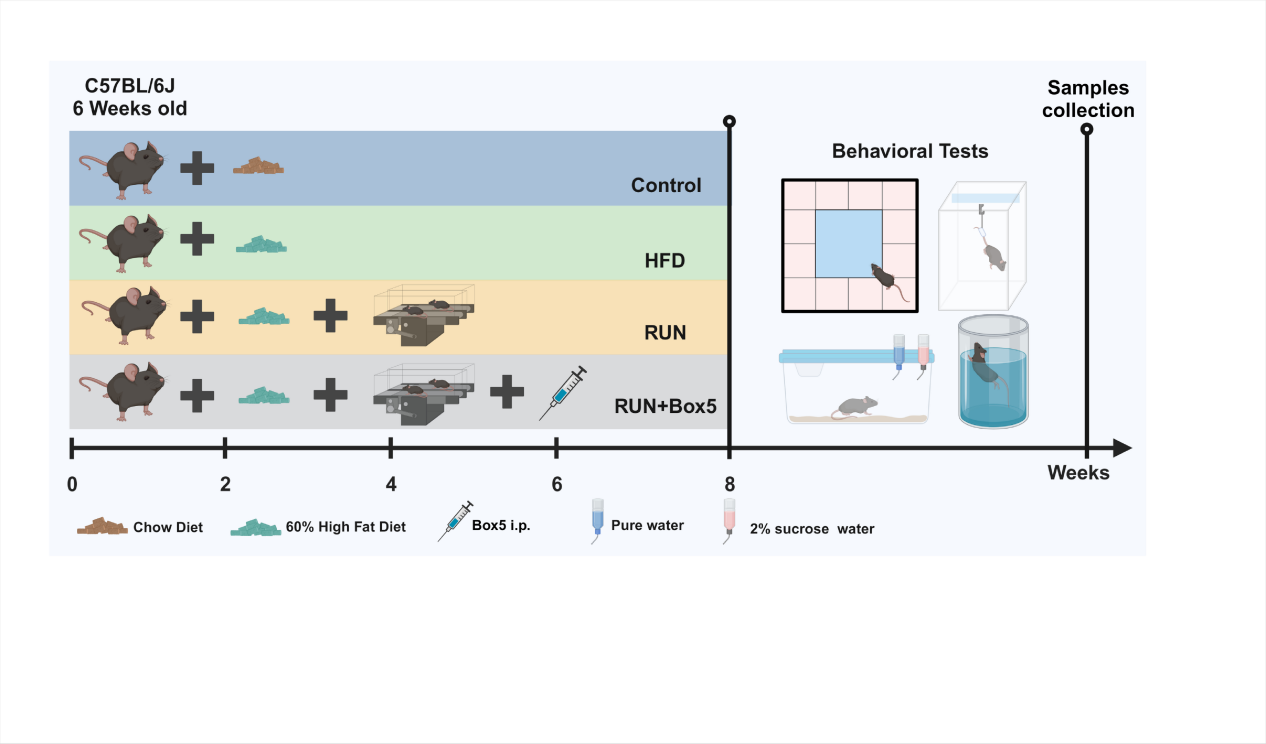


**Fig. S6. Study timeline 2.** Control: The group given standard chow. HFD: The group given a high-fat diet (60% kcal from fat). RUN: The group given a high-fat diet (60% kcal from fat) and exercised on treadmill equipment at the same time. RUN+Box5: The group treated with Box5. (Created with BioRender.com)


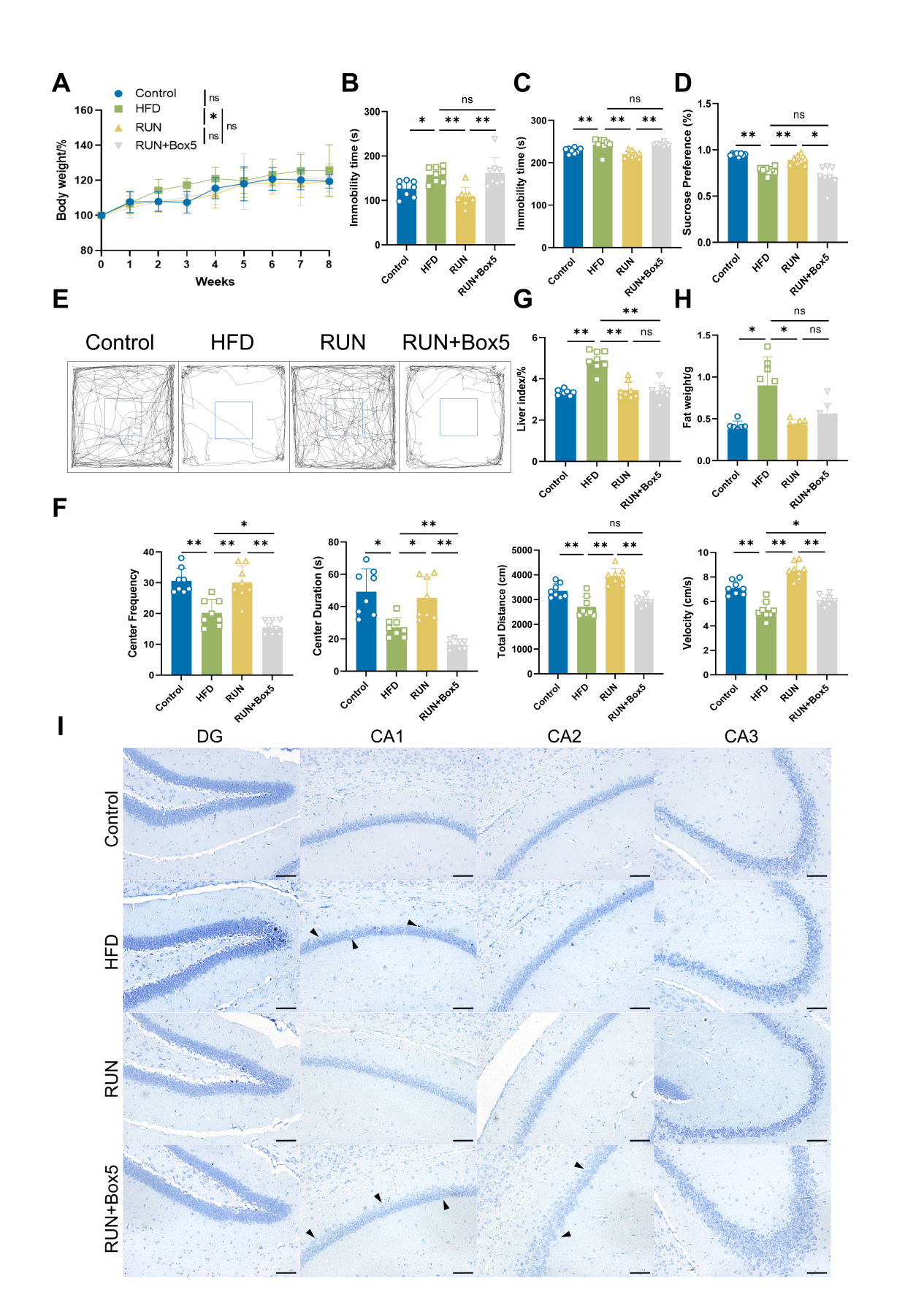


**Fig. S7. Inhibition of Wnt5a counteracts the ameliorative effect of exercise on depressive-like behavior in HFD-fed mice.** (A) Body weight (n = 12). (B) Immobility time in the tail suspension test (TST) (n = 8). (C) Immobility time in the forced swimming test (FST) (n = 8). (D) Sucrose preference test (n = 8). (E) Track visualization image of OFT. (F) Comprehensive behaviors of mice in the open field test (OFT) (n = 8). (G) Liver index of different group (n = 8). (H) Epididymal fat weight of different group (n = 8). (I) Arrangement and number of hippocampal neurons in different groups (bar = 100 μm). All the results are presented as the means ± standard deviations (SDs) with statistical significance (**P* < 0.05, ***P* < 0.01, ns: *P* > 0.05).

**
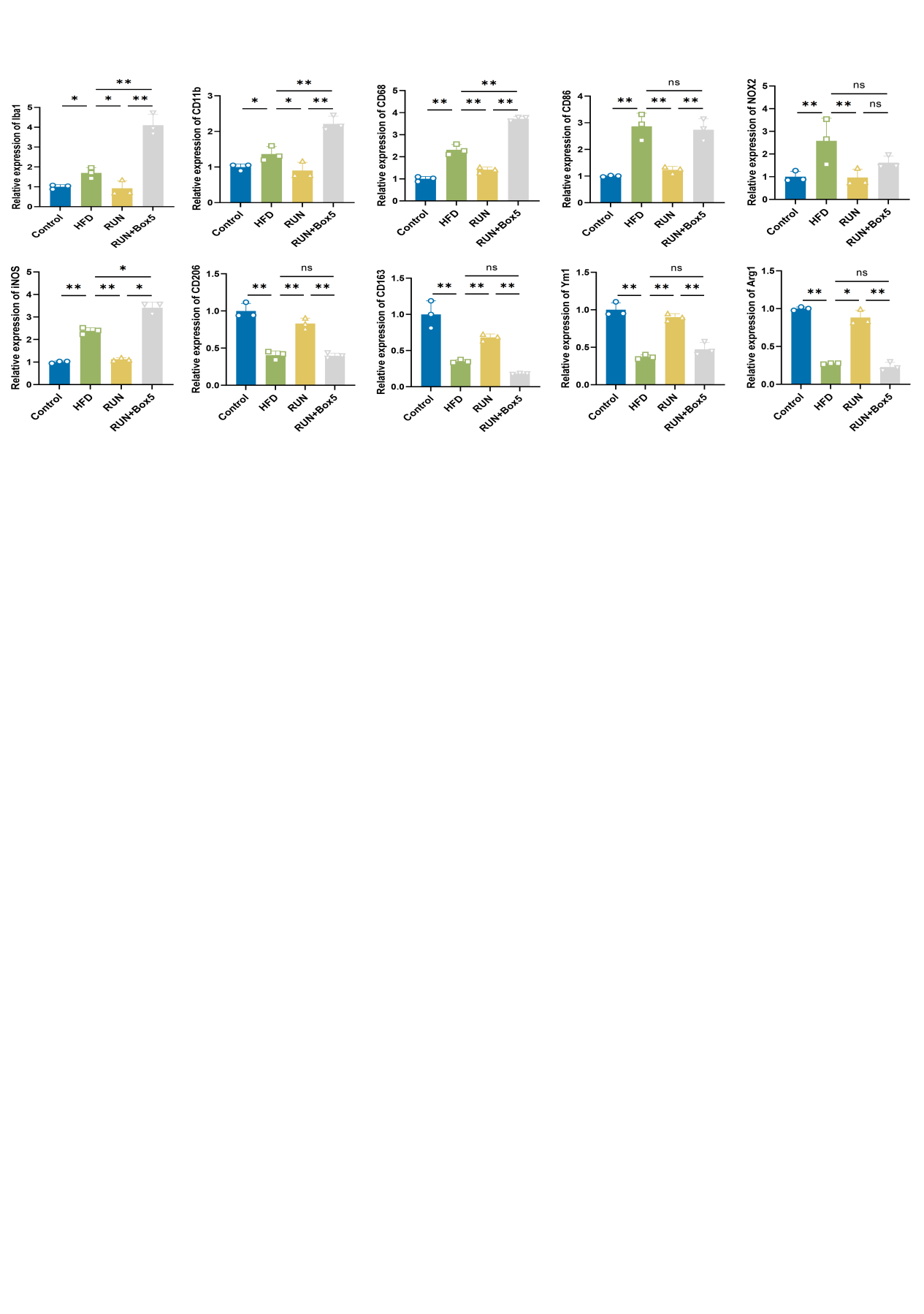
**

**Fig. S8. Relative expression of microglial cell surface markers (n = 3).** All the results are presented as the means ± standard deviations (SDs) with statistical significance (**P* < 0.05, ***P* < 0.01, ns: *P* > 0.05).
