# Supplementary material for: Regular exercise ameliorates high-fat diet-induced depressive-like behaviors by activating hippocampal neuronal autophagy and enhancing synaptic plasticity
Source: Cell Death Dis. 2024 Oct 10;15(10):737. doi: 10.1038/s41419-024-07132-4 (PMC11467387; doi:10.1038/s41419-024-07132-4)

Original WB bands in this study

Figure 3C

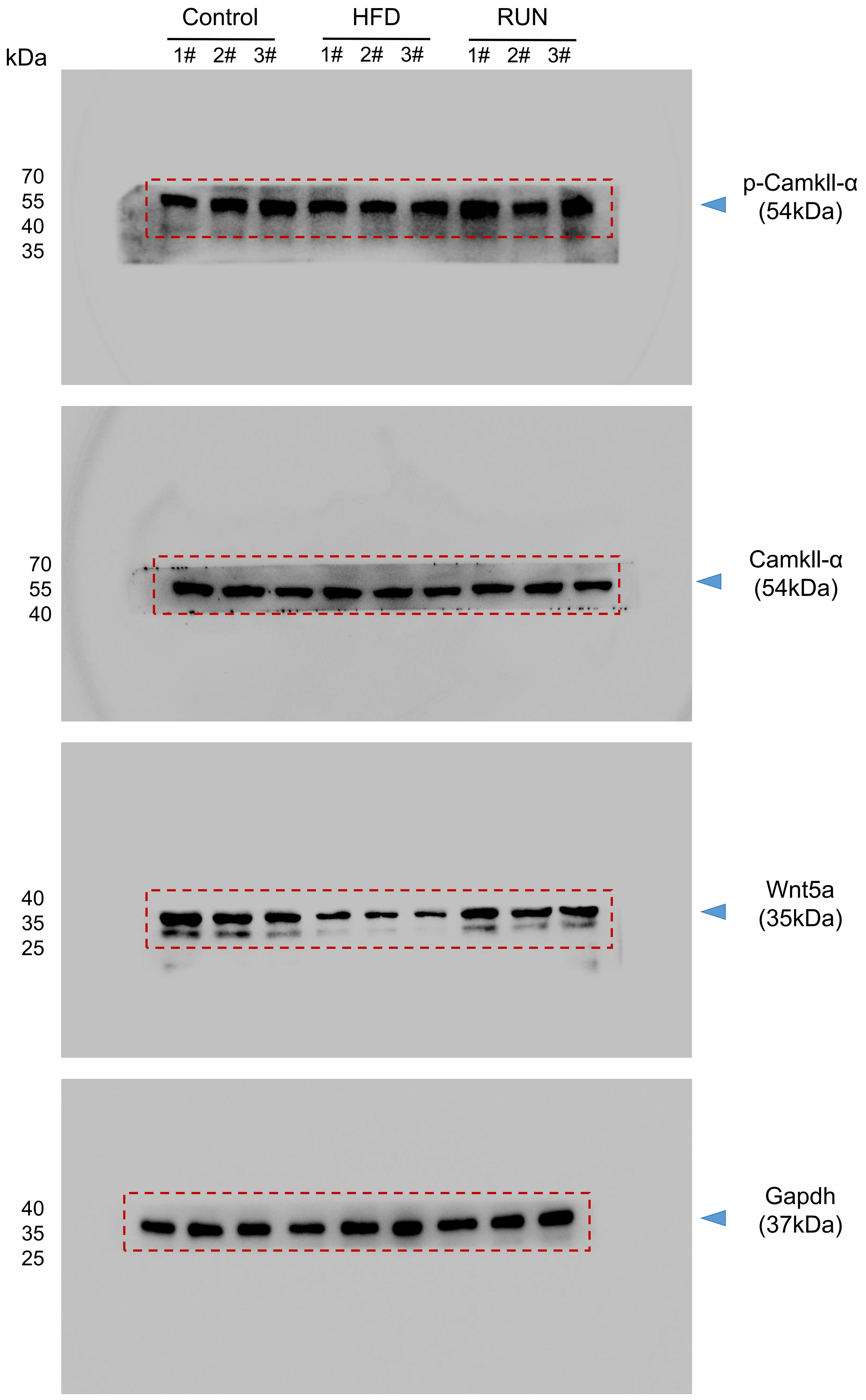

Original WB bands in this study

Figure 4C

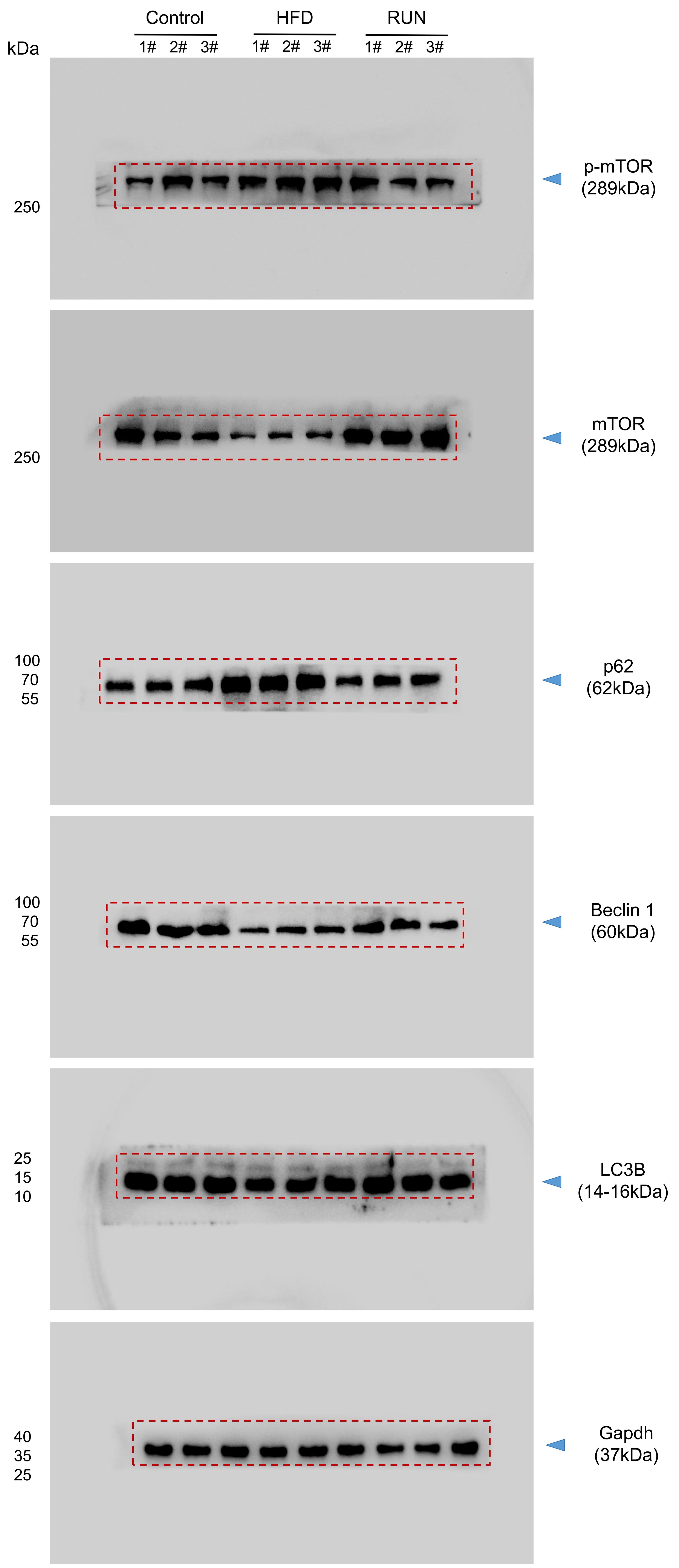

Figure 4J

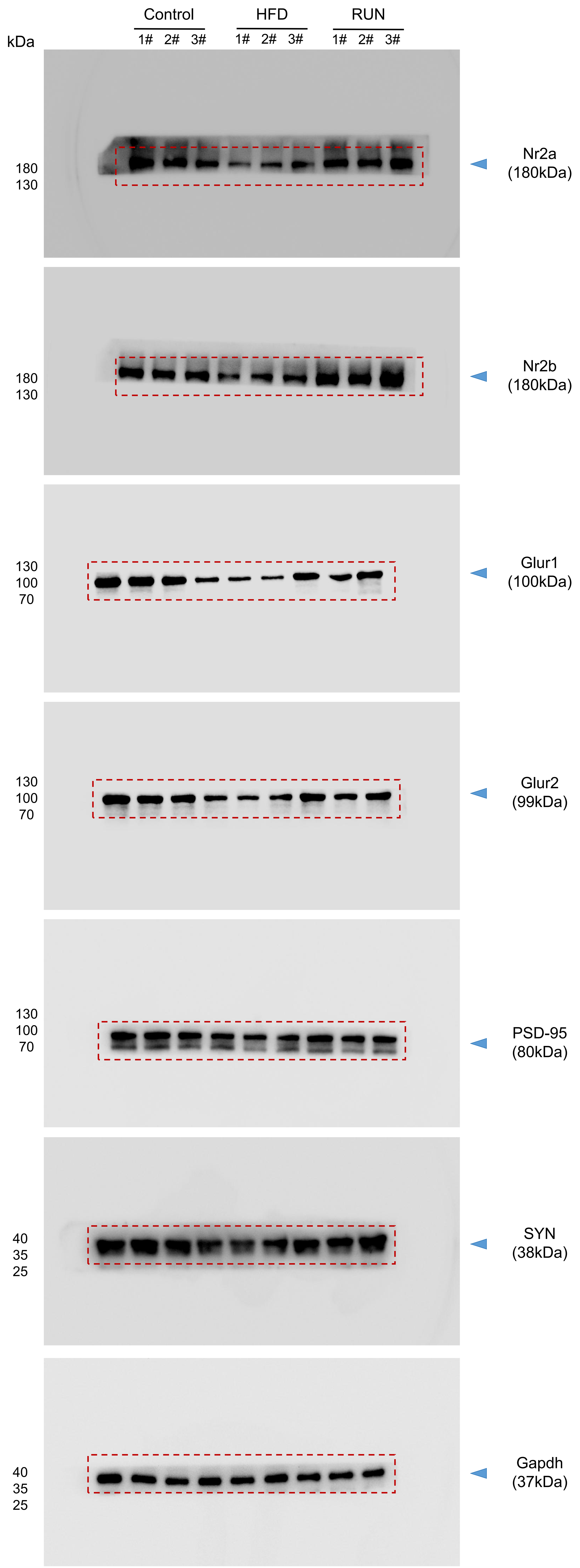

Original WB bands in this study

Figure 5D

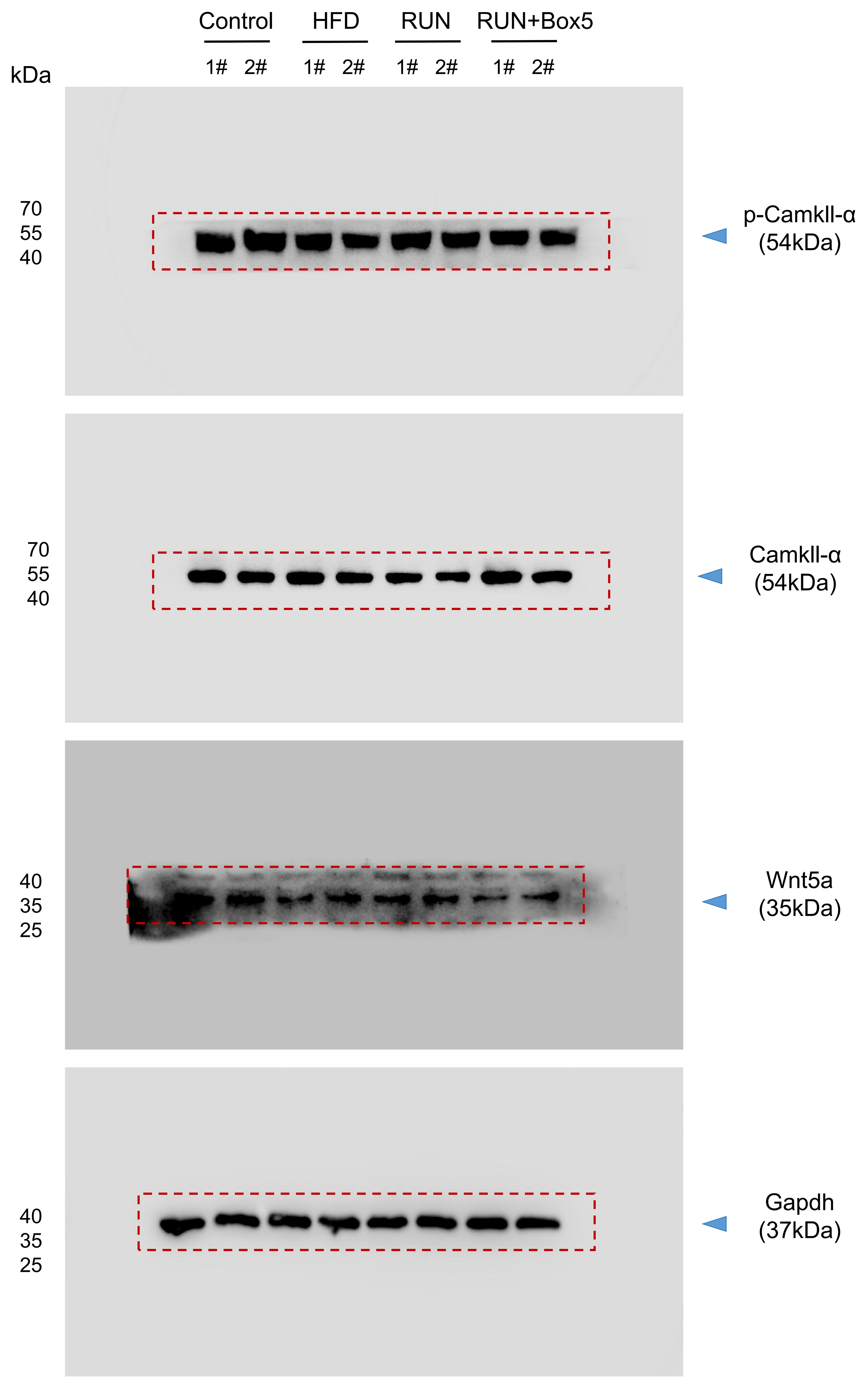

Figure 5J

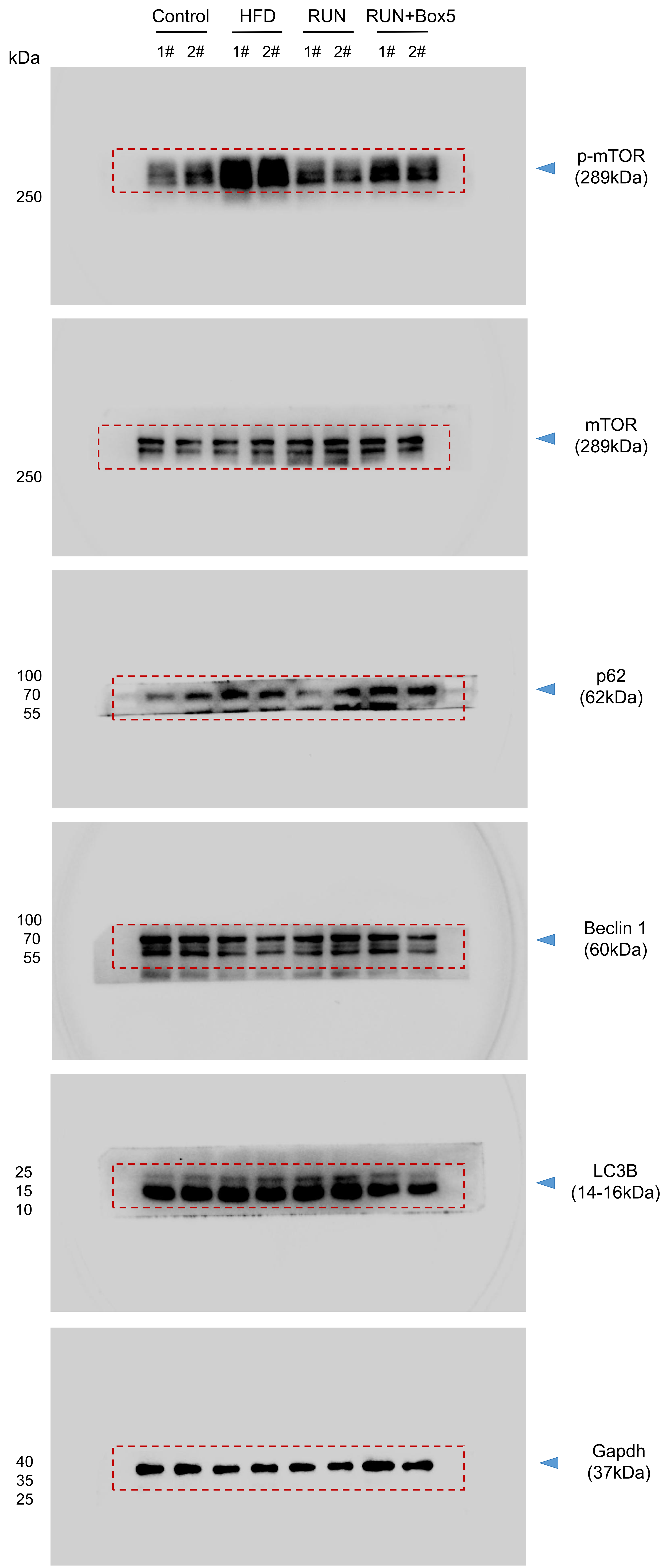

Original WB bands in this study

Figure 6E

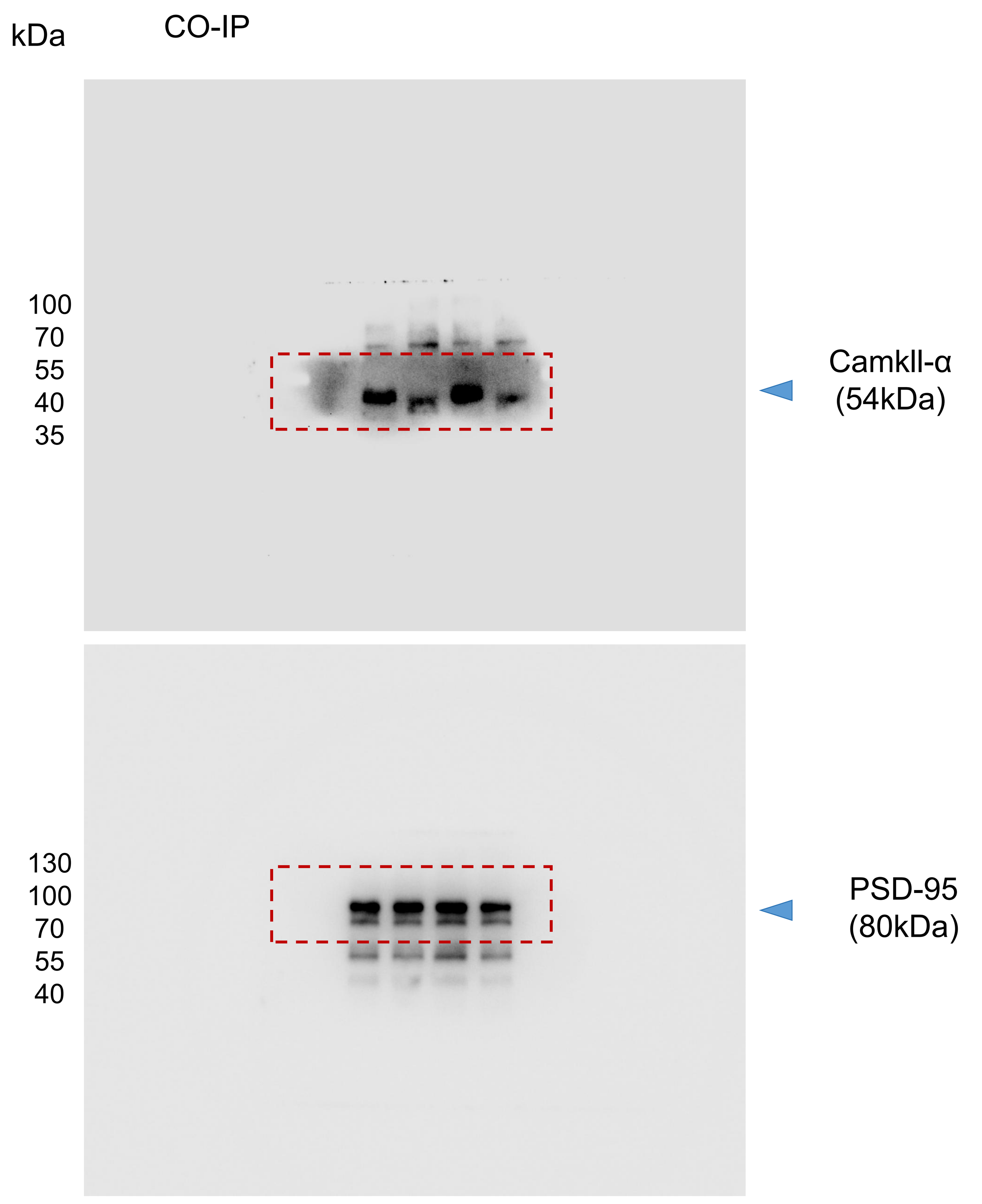

The order of protein samples from left to right are Control, HFD, RUN, RUN+Box5.

Figure 6F

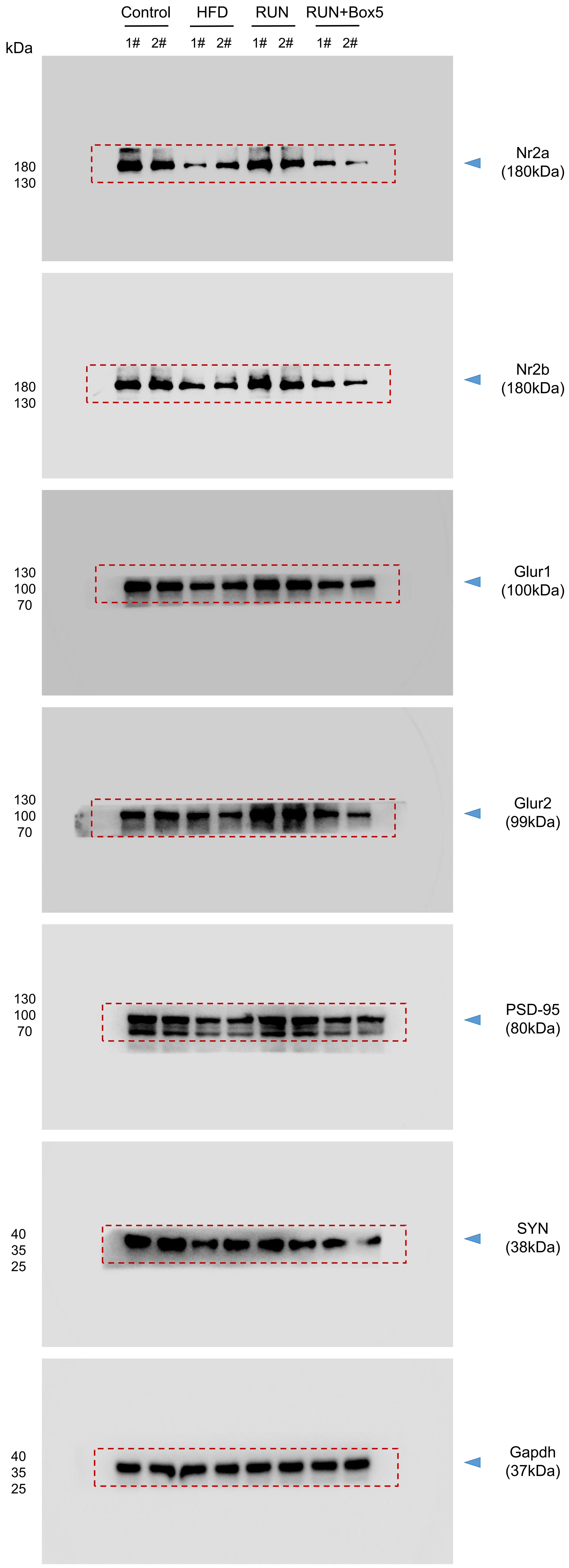

Supplement: Supplementary file 2 — Original Western Blots [file 41419_2024_7132_MOESM2_ESM.pdf]
